# MYCT1 controls environmental sensing in human haematopoietic stem cells

**DOI:** 10.1038/s41586-024-07478-x

**Published:** 2024-06-05

**Authors:** Júlia Aguadé-Gorgorió, Yasaman Jami-Alahmadi, Vincenzo Calvanese, Maya Kardouh, Iman Fares, Haley Johnson, Valerie Rezek, Feiyang Ma, Mattias Magnusson, Yanling Wang, Juliana E. Shin, Karina J. Nance, Helen S. Goodridge, Simone Liebscher, Katja Schenke-Layland, Gay M. Crooks, James A. Wohlschlegel, Hanna K. A. Mikkola

**Affiliations:** 1 Department of Molecular, Cell and Developmental Biology, University of California Los Angeles, Los Angeles, CA USA; 2 Eli and Edythe Broad Center for Regenerative Medicine and Stem Cell Research, University of California Los Angeles, Los Angeles, CA USA; 3 Department of Biological Chemistry, University of California Los Angeles, Los Angeles, CA USA; 4 Laboratory for Molecular Cell Biology, University College London, London, UK; 5 Josep Carreras Leukaemia Research Institute, Barcelona, Spain; 6 David Geffen School of Medicine at UCLA, Los Angeles, CA USA; 7 UCLA AIDS Institute, University of California Los Angeles, Los Angeles, CA USA; 8 Institute for Genomics and Proteomics, University of California Los Angeles, Los Angeles, CA USA; 9 Division of Molecular Medicine and Gene Therapy, Lund Stem Cell Center, Lund University, Lund, Sweden; 10 Board of Governors Regenerative Medicine Institute, Cedars-Sinai Medical Center, Los Angeles, CA USA; 11 Institute of Biomedical Engineering, Department for Medical Technologies and Regenerative Medicine, Eberhard Karls University, Tübingen, Germany; 12 NMI Natural and Medical Sciences Institute at the University Tübingen, Reutlingen, Germany; 13 Jonsson Comprehensive Cancer Center, University of California Los Angeles, Los Angeles, CA USA; 14 Department of Pathology and Laboratory Medicine, David Geffen School of Medicine, University of California Los Angeles, Los Angeles, CA USA; 15 Molecular Biology Institute, University of California Los Angeles, Los Angeles, CA USA; 16 Present Address: Pfizer, Cambridge, MA USA; 17 Present Address: Oakland University William Beaumont School of Medicine, Rochester, MI USA; 18 Present Address: Kite Pharma, Santa Monica, CA USA; 19 Present Address: Department of Laboratory Medicine, University of California San Francisco, San Francisco, CA USA; 20 Present Address: Amgen, Thousand Oaks, CA USA; 21 Present Address: David Geffen School of Medicine at UCLA, Los Angeles, CA USA

**Keywords:** Haematopoietic stem cells, Self-renewal, Stem-cell niche, Endocytosis, Growth factor signalling

## Abstract

The processes that govern human haematopoietic stem cell (HSC) self-renewal and engraftment are poorly understood and challenging to recapitulate in culture to reliably expand functional HSCs^1–3^. Here we identify MYC target 1 (MYCT1; also known as MTLC) as a crucial human HSC regulator that moderates endocytosis and environmental sensing in HSCs. MYCT1 is selectively expressed in undifferentiated human haematopoietic stem and progenitor cells (HSPCs) and endothelial cells but becomes markedly downregulated during HSC culture. Lentivirus-mediated knockdown of *MYCT1* prevented human fetal liver and cord blood (CB) HSPC expansion and engraftment. By contrast, restoring MYCT1 expression improved the expansion and engraftment of cultured CB HSPCs. Single-cell RNA sequencing of human CB HSPCs in which *MYCT1* was knocked down or overexpressed revealed that MYCT1 governs important regulatory programmes and cellular properties essential for HSC stemness, such as ETS factor expression and low mitochondrial activity. MYCT1 is localized in the endosomal membrane in HSPCs and interacts with vesicle trafficking regulators and signalling machinery. MYCT1 loss in HSPCs led to excessive endocytosis and hyperactive signalling responses, whereas restoring MYCT1 expression balanced culture-induced endocytosis and dysregulated signalling. Moreover, sorting cultured CB HSPCs on the basis of lowest endocytosis rate identified HSPCs with preserved MYCT1 expression and MYCT1-regulated HSC stemness programmes. Our work identifies MYCT1-moderated endocytosis and environmental sensing as essential regulatory mechanisms required to preserve human HSC stemness. Our data also pinpoint silencing of MYCT1 as a cell-culture-induced vulnerability that compromises human HSC expansion.

## Abstract (web summary)

MYCT1 is identified as a crucial regulator of cellular properties essential for the stemness of human haematopoietic stem cells, a finding that may help improve successful in vitro expansion of these cells for therapeutics.

## Main

HSCs sustain blood formation throughout life owing to their ability to respond to microenvironmental cues to balance self-renewal and differentiation. HSCs can provide life-saving therapy for patients with haematological malignancies and patients with inherited blood disorders. But access to HSC transplantation is limited by the difficulty in finding immune-compatible bone marrow (BM) donors and the low quantity of HSCs in cord blood (CB)^1–3^. Thus, it has been a long-standing goal to optimize the cell culture environment to expand human HSCs ex vivo^4,5^. However, transfer of human HSCs from their niche to culture results in extensive changes in transcriptome, epigenome, signalling, metabolism, proteostasis and other cellular functions that severely compromise HSC function^4,6–11^.

The diverse approaches available to recapitulate human HSC self-renewal ex vivo include co-culture with niche cells such as BM mesenchymal stromal cells^9,12^ or endothelial cells (ECs)^13^, and 3D cultures within a hydrophilic matrix^11^. Other methods include supplementation of HSC cytokines with HSC-supportive small molecules such as SR1 (ref. ^14^) or UM171 (ref. ^15^), and albumin-free conditions that replace cytokines with chemical agonists^16^. Moreover, transcriptional regulators that can promote human HSC expansion in culture, such as MLLT3 (ref. ^10^) and MSI2 (ref. ^17^), have been discovered and functionally validated following transplantation. Another goal is to identify molecular markers that indicate the preservation of HSC properties. Recent advances include more specific surface markers for cultured human HSCs, such as EPCR^18^, ITGA3 (ref. ^19^) and RET^20^, and signature genes in the HSC transcriptome (for example, *MLLT3* (ref. ^10^), *HLF*^21,22^ and *MECOM*^23^). Desirable cellular characteristics such as low mitochondrial activity and oxidative phosphorylation (OXPHOS)^24,25^, balanced proteostasis^26,27^ or active lysosomal function^28^ are also goals. Nevertheless, the expansion of human haematopoietic cells that retain a HSC immunophenotype does not directly translate into repopulation ability following transplantation^6,7^. Maximizing the yield of functional human HSCs in culture requires a better understanding of the processes that govern HSC self-renewal and engraftment ability and why they become compromised in culture. Here we identify MYCT1 as a pivotal human HSC regulator that controls endocytosis in HSCs and thereby moderates how HSCs sense microenvironmental signals. Our study links MYCT1 expression and low endocytosis rate as molecular markers for sustained human HSC function. Our results also pinpoint silencing of MYCT1 expression in cultured human HSCs as a vulnerability that compromises the function of ex vivo expanded human HSCs.

## Cultured human HSCs lose MYCT1 expression

**Fig. 1:**
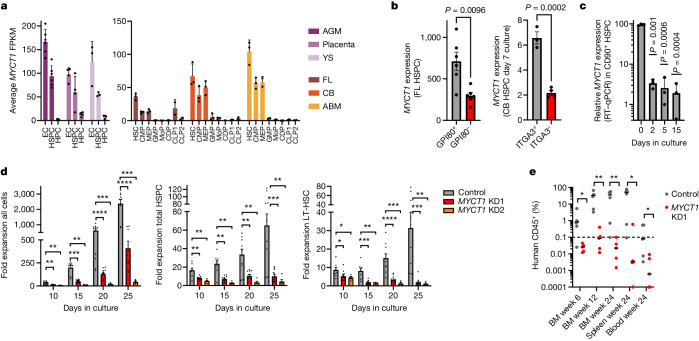
MYCT1 is crucial for human HSPC ex vivo expansion and engraftment. **a**, *MYCT1* expression from RNA-seq on sorted populations from human 5–6-week AGM, placenta, yolk sac (YS), second trimester FL, CB and adult bone marrow (ABM). *n* = 6 (AGM), *n* = 4 (placenta) or *n* = 3 (YS, FL, CB, ABM) donors per tissue, mean ± s.e.m. FPKM, fragments per kilobase of mappable length and million counts. CMP, common myeloid progenitor; MEP, megakaryocyte erythroid progenitor; GMP, granulocyte macrophage progenitor; CDP, common dendritic cell progenitor; CLP, common lymphoid progenitor. **b**, MYCT1 expression in human FL GPI80^+^ and GPI80^–^ HSPC (CD34^+^CD38^–^CD90^+^) subsets^30^, and UM171-expanded CB ITGA3^+^ and ITGA3^–^ HSPC (CD90^+^EPCR^+^CD133^+^CD34^+^CD45RA^–^) subsets^19^. *n* = 3 and 4 replicates, mean ± s.e.m., two-tailed paired and unpaired *t*-test, respectively. **c**, Relative *MYCT1* expression by qPCR with reverse transcription (RT–qPCR) in CB HSPCs (CD34^+^CD38^–^CD90^+^) sorted before and after culture. *n* = 3 experiments, mean ± s.e.m., two-tailed paired *t*-test. **d**, Fold ex vivo expansion of all cells, total HSPCs (CD34^+^CD38^–^CD90^+^CD45RA^–^) and immunophenotypic long-term HSCs (LT-HSCs; CD34^+^CD38^–^CD90^+^CD45RA^–^EPCR^+^ITGA3^+^) from control or *MYCT1* KD CB. *n* = 8 (control), *n* = 12 (KD1), *n* = 5 (KD2) replicates from 4 independent experiments, mean ± s.e.m., two-tailed Mann–Whitney test. Left to right: all cells KD1: *P* = 0.041, *P* = 0.000016, *P* = 0.000059, *P* = 0.000037; KD2: *P* = 0.0016, *P* = 0.0016, *P* = 0.0007, *P* = 0.0007; total HSPC KD1: *P* = 0.0055, *P* = 0.0022, *P* = 0.0044, *P* = 0.0004; KD2: *P* = 0.0062, *P* = 0.0031, *P* = 0.0027, *P* = 0.0027; LT-HSC KD1: *P* = 0.0252, *P* = 0.0002, *P* = 0.000022, *P* = 0.0008; LT-HSC KD2: *P* = 0.0186, *P* = 0.0016, *P* = 0.0007, *P* = 0.0083. **e**, Quantification of human haematopoietic engraftment (human CD45^+^) in NSG mice transplanted with equal numbers (5,000) of sorted control or *MYCT1* KD CB HSPCs. *n* = 6 (control) and *n* = 5 (*MYCT1* KD) mice per group, mean ± s.e.m., two-tailed Mann–Whitney test. Left to right: *P* = 0.0303, *P* = 0.0043, *P* = 0.0087, *P* = 0.0173, *P* = 0.0303. Source Data

**Extended Data Fig. 1:**
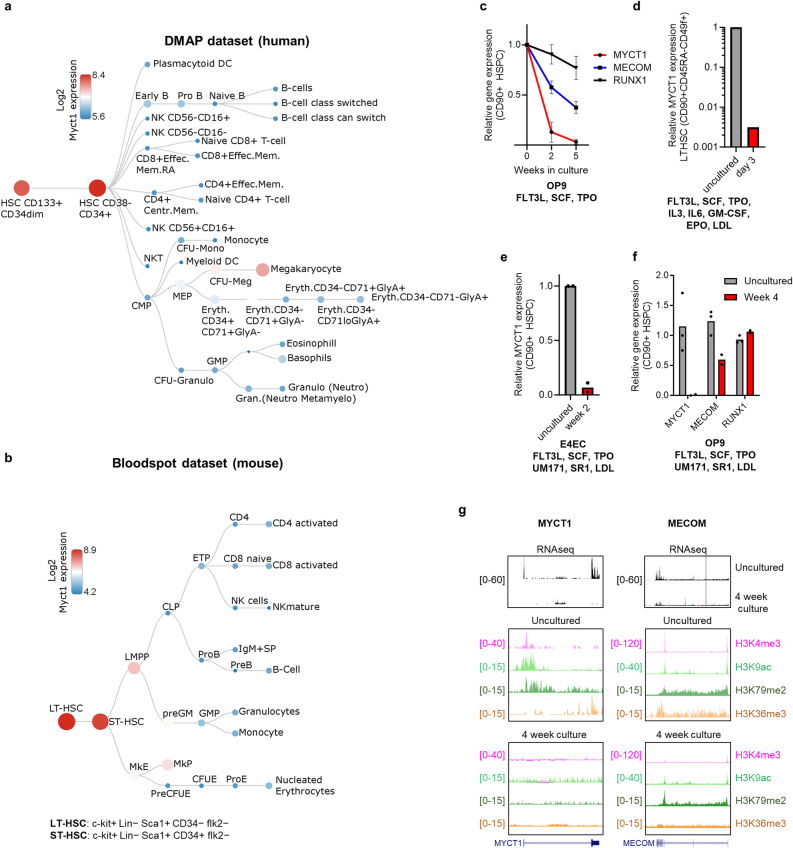
Silencing of MYCT1 expression in cultured human HSPCs. **a** MYCT1 expression in human sorted hematopoietic populations from the DMAP dataset^29,31^. **b** Myct1 expression in mouse hematopoietic populations from Bloodspot^31^. **c-g** Relative expression of MYCT1 and other HSC regulatory genes in sorted FL or CB HSPCs that were isolated freshly and after culture in different conditions. **c** Microarray analysis of CD34^+^CD38^−^CD90^+^FL HSPCs co-cultured with OP9 stroma supplemented with cytokines as indicated^9^. Mean±s.e.m. of all available microarray probes for MYCT1 (2), MECOM (5), and RUNX1 (10) from n = 3 (uncultured and week 2) or n = 2 (week 5) independent experiments. **d** RNAseq analysis of CD34^+^CD38^−^CD90^+^CD45RA^-^CD49f^+^ CB LT-HSPC cultured without stroma as indicated^28^. **e** RT-qPCR of CD34^+^CD38^−^CD90^+^ CB HSPCs co-cultured with E4EC^13^ as indicated. n = 2 experiments, mean and individual data points. **f,g** Gene expression (RNAseq) and UCSC genome browser tracks of MYCT1 and MECOM genomic regions showing RNA seq and Chip-seq of histone marks in CD34^+^CD38^−^CD90^+^ FL HSPCs isolated freshly or co-cultured with OP9 stroma as indicated^10^. Gene expression is from n = 3 (uncultured) or n = 2 (week 4) experiments, mean with individual data points. Source Data

First, we aimed to uncover regulators of human HSC self-renewal that underlie the compromised function of cultured human HSCs. To that end, we analysed RNA sequencing (RNA-seq) datasets of human developmental and postnatal haematopoietic tissues and identified HSC-enriched genes that become downregulated during culture. MYCT1 was selectively expressed in ECs and in undifferentiated HSPCs in all human haematopoietic tissues and developmental stages. These included 5–6-week aorta–gonad–mesonephros (AGM) region, placenta, yolk sac, second trimester fetal liver (FL), CB, adult BM (Fig. 1a) and megakaryocytes^29^ (Extended Data Fig. 1a). Moreover, MYCT1 levels were highest within the HSPC fractions enriched for the transplantable, self-renewing human HSCs in FL (GPI80^+^ fraction^30^) and in ex vivo-expanded CB HSPCs (ITGA3^+^ fraction^19^) (Fig. 1b). *Myct1* expression is also enriched in mouse long-term HSCs (LT-HSCs; KIT^+^LIN^–^SCA1^+^CD34^–^FLK2^–^) compared with short-term HSCs (ST-HSCs; KIT^+^LIN^–^SCA1^+^CD34^+^FLK2^–^) and differentiated progenitors^31^ (Extended Data Fig. 1b). Evaluation of microarray and RNA-seq datasets of human HSPCs cultured in various conditions and treated with cytokines, small molecules and/or niche cells and quantitative PCR (qPCR) analysis of cultured CB HSPCs revealed that MYCT1 expression is highly sensitive to exposure to culture^9,10,28^ (Fig. 1c and Extended Data Fig. 1c–f). Moreover, prolonged HSPC culture led to silencing of the active epigenetic marks at the transcription start site and gene body associated with MYCT1 expression in human HSPCs (Extended Data Fig. 1g). These data tightly link MYCT1 expression with HSC self-renewal and engraftment ability.

## MYCT1 loss disrupts human HSC function

**Extended Data Fig. 2:**
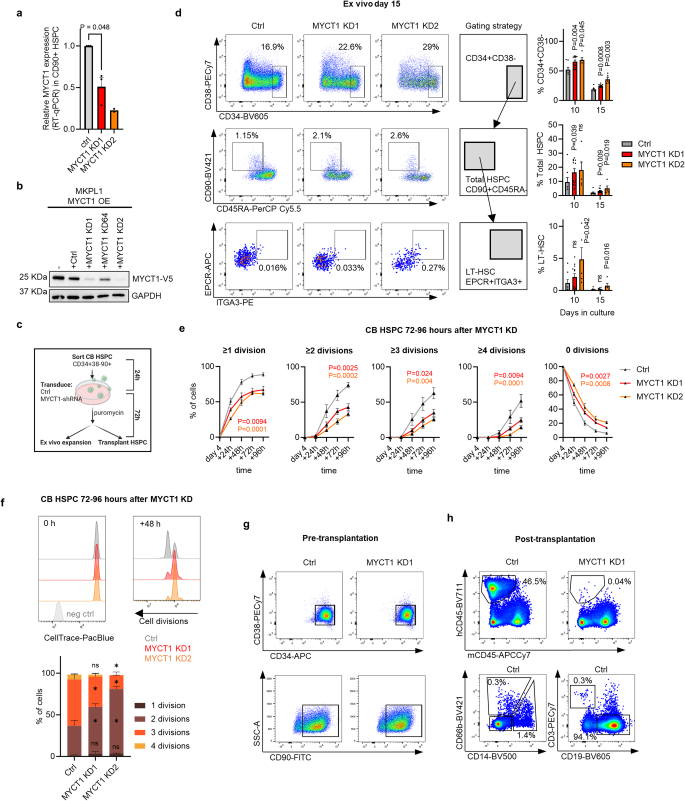
Validation of MYCT1 knockdown and its effects in cord blood HSPCs. **a,b** Validating MYCT1 knockdown. **a** Relative MYCT1 expression by RT-qPCR in control or MYCT1 knockdown (KD) sorted CB HSPCs (CD34^+^CD38^−^CD90^+^) 72 h post-transduction. Mean±s.e.m, two-tailed paired t-test, n = 3 (KD1) or mean, n = 2 (KD2) independent experiments. **b** Western blot in MYCT1-V5 overexpressing (OE) MKPL1 cells after transduction with control or MYCT1 KD lentiviral vectors. n = 1 experiment. GAPDH is a sample processing control. For gel source data see Supplementary Fig. 2. **c** Experimental outline to evaluate the effects of MYCT1 KD on CB HSPC ex vivo expansion and engraftment. **d** Representative FACS plots, gating strategy, and quantification of immunophenotypic HSPC/HPC fractions from control or MYCT1 KD CB HSPCs after 15 days in culture. Percentage of cells in each population within the total cells is indicated. Corresponds to quantifications in Fig. 1d. n = 8 (Ctrl), n = 12 (KD1), n = 5 (KD2) replicates from 4 independent experiments, mean±s.e.m., two-tailed Mann–Whitney test. **e,f** Quantifying proliferation in HSPCs after MYCT1 KD. **e** Percentage of HSPCs (CD34^+^CD38^–^CD90^+^) that have undergone at least 1 to 4 divisions, or no divisions, for every 24 h elapsed since plating. n = 4 independent experiments, mean±s.e.m, two-tailed t-test. *P* values at 96 h timepoint are depicted. **f** Representative FACS histograms and quantification of proliferation by dye dilution (CellTrace) in control or MYCT1 KD CB HSPCs (CD34^+^CD38^−^CD90^+^) at time of labelling (72-96 h post-transduction, indicated as 0 h) and after 48 additional hours. n = 4 (KD1) and n = 3 (KD2) independent experiments, mean±s.e.m, two-tailed Mann-Whitney t-test. For KD1 *P = 0.0317, for KD2 *P = 0.0159. **g** FACS plots showing control or MYCT1 KD CB HSPCs sorted for transplantation (CD34^+^CD38^−^CD90^+^) 72 h post-transduction. **h** Representative FACS plots of human hematopoietic engraftment (hCD45^+^) and differentiated populations in mice transplanted with equal number (5,000) of sorted control or MYCT1 KD CB HSPCs. In FACS plots, percentage within total hCD45^+^ cells indicated. Schematic in **c** was created with BioRender (https://www.biorender.com). Source Data

**Extended Data Fig. 3:**
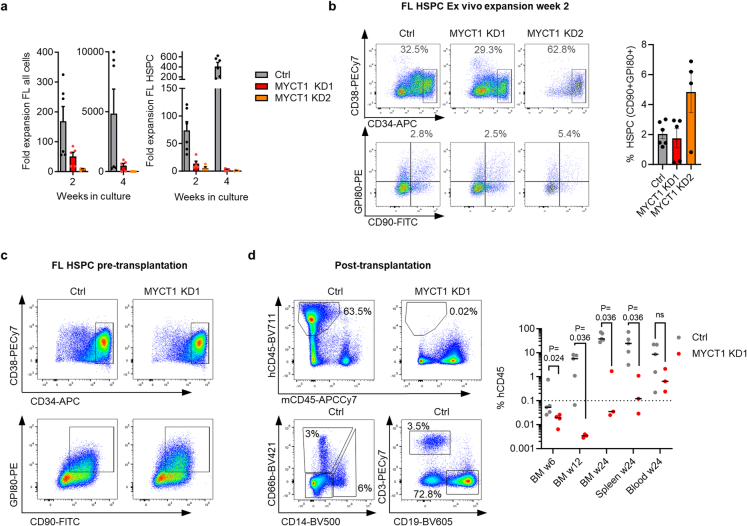
Documentation of the effects of MYCT1 knockdown on human foetal liver HSPC expansion and engraftment. **a,b** Quantifying expansion of foetal liver (FL) HSPC after MYCT1 KD. Fold expansion (**a**), representative FACS plots and percentage (**b**) of all live cells and immunophenotypic self-renewing HSCs (CD34^+^CD38^−^CD90^+^GPI80^+^) from control or MYCT1 KD FL. n = 6 (ctrl), n = 5 (KD1) and n = 4 (KD2) replicates from 2 independent experiments, mean±s.e.m. **c,d** Determining the transplantation ability of MYCT1 KD FL HSPCs. **c** FACS plots showing control or MYCT1 KD CB HSPCs sorted for transplantation 72 h after transduction. The gate indicates the population (CD34^+^CD38^−^CD90^+^GPI80^+^) that was sorted and transplanted. 10,000 sorted control or KD HSPCs were transplanted per mouse. **d** Representative FACS plots and quantification of human hematopoietic engraftment (hCD45^+^) and multilineage differentiation in NSG mice 24 weeks after transplantation. In FACS plots, percentage within total hCD45^+^ cells indicated. Quantification shows bone marrow at 6, 12 and 24 weeks after transplantation, and spleen and blood 24 weeks after transplantation. n = 5 (control) and n = 4 (MYCT1 KD) mice per group, mean±s.e.m., two-tailed Mann–Whitney test. Source Data

To understand MYCT1 function in human CB HSPCs, we performed lentiviral short hairpin RNA (shRNA)-mediated knockdown (KD) using two different *MYCT1* shRNAs (Extended Data Fig. 2a–c). *MYCT1* KD severely halted the expansion of CB LT-HSCs (CD34^+^CD38^–^CD90^+^CD45RA^–^EPCR^+^ITGA3^+^), total HSPCs (CD34^+^CD38^–^CD90^+^CD45RA^–^) and their progeny, but did not ablate them (Fig. 1d and Extended Data Fig. 2d). Instead, *MYCT1* KD slowed down HSPC cell division kinetics, which indicated a defect in proliferative potential (Extended Data Fig. 2e,f). When equal numbers of sorted CB HSPCs (CD34^+^CD38^–^CD90^+^) were transplanted into NSG mice, *MYCT1* KD completely abolished HSC engraftment ability, whereas control cells showed sustained multilineage engraftment (Fig. 1e, Extended Data Fig. 2g,h and Supplementary Table 1). *MYCT1* KD in second trimester FL HSPCs also prevented fetal HSC (CD34^+^CD38^–^CD90^+^GPI80^+^) expansion and generation of progeny in culture, and abrogated transplantation ability (Extended Data Fig. 3a–d and Supplementary Table 1). These data identify MYCT1 as a previously unknown human HSC regulator that is important for culture expansion and engraftment across HSC ontogeny.

## MYCT1 maintains HSC stemness programmes

**Fig. 2:**
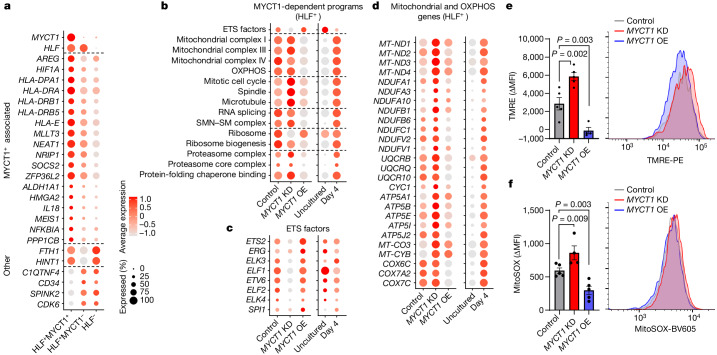
MYCT1 governs regulatory programmes associated with human HSC functional competence. **a**, scRNA-seq dot plot documenting the expression of selected human HSC-enriched genes from multiple datasets^18,33–36^, as well as the HSC activation marker CDK6, that are significantly differentially expressed in uncultured CB HSPCs (CD34^+^CD38^–^CD90^+^) selected on the basis of HLF and the presence or absence of MYCT1 expression. **b**, scRNA-seq dot plot depicting the scores for the differentially regulated functional categories comparing HLF^+^ HSCs from cultured control, *MYCT1* KD and *MYCT1* OE HSPCs. Uncultured and cultured HLF^+^ HSCs are also compared. **c**, Dot plot depicting gene expression of ETS factors. **d**, Dot plot depicting gene expression of selected mitochondrial and OXPHOS genes. **e**,**f**, Quantification (left) and representative FACS plots (right) of mitochondrial membrane potential (TMRE) (**e**) and mitochondrial reactive oxygen species (MitoSOX) (**f**) in control, *MYCT1* KD and *MYCT1* OE HSPCs 72 h after transduction. Delta median fluorescence intensity (ΔMFI) is between the GFP^+^ cells and the GFP^–^ cells within the same sample. *n* = 5 replicates from 3 independent experiments, mean ± s.e.m., two-tailed unpaired *t*-test. Source Data

**Extended Data Fig. 4:**
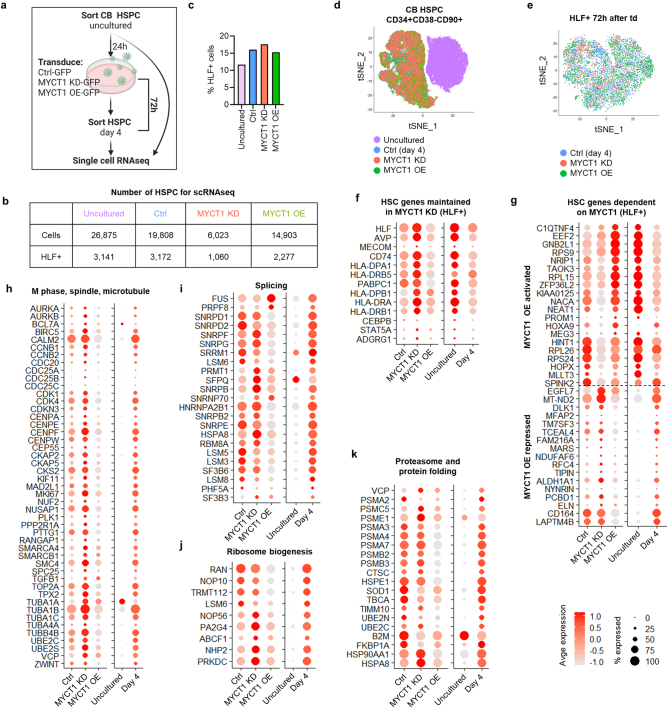
Analysis of MYCT1 dependent programs in cultured human CB HSCs. **a** Experimental outline for scRNAseq analysis evaluating the effects of MYCT1 KD and MYCT1 OE in CB HSPCs 72 h after transduction (4 days in culture). CB HSPCs (CD34^+^CD38^−^CD90^+^) were isolated freshly or 72 h after transduction. **b** Number of total HSPCs and HLF^+^ cells sequenced for scRNAseq for each sample. **c** Percentage of HLF^+^ HSC among uncultured and cultured control, MYCT KD and OE HSPC. **d,e** TSNE plots showing all the sequenced HSPCs (**d**), or the selected HLF^+^ cells 72 h after transduction (**e**). **f,g** Dot plot depicting gene expression of selected human HSC signature genes^18,33–36^ that are significantly differentially expressed in HLF^+^ HSCs from MYCT1 KD and/or MYCT1 OE compared to control. Comparison of uncultured and cultured HLF^+^ HSCs is also shown. **h-k** scRNAseq dot plots depicting expression for selected genes in the programs found to be differentially regulated by MYCT1 KD or OE in HLF^+^ HSC (see Fig. 2b). n = 1 experiment with the indicated number of single cells sequenced. Schematic in **a** was created with BioRender (https://www.biorender.com). Source Data

**Extended Data Fig. 5:**
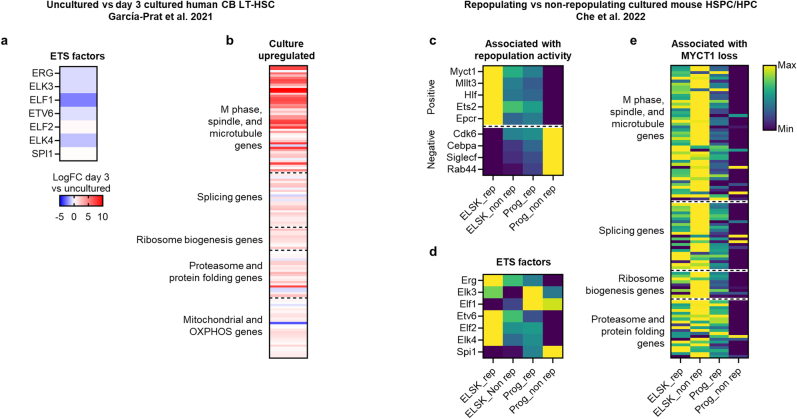
Analysis of MYCT1 associated programs in independent human and mouse datasets reflecting different levels of HSC competence. **a,b** Evaluating the expression of MYCT1 associated “stemness” programmes in uncultured compared to 3 day cultured CB LT-HSC (CD34^+^CD38^−^CD90^+^CD45RA^−^CD49f^+^)^28^. Heatmaps depict Log Fold change. **c-e** Evaluating the expression of Myct1 and MYCT1 associated human HSPC “stemness” programmes in mouse repopulating and non-repopulating HSC and progenitors in PVA-cultured mouse HSPCs^41^. Mouse sorted immunophenotypic HSPCs (ELSK: EPCR^+^Lin^−^Sca-1^+^C-kit^−^) which were able repopulate mice upon transplantation (ELSK_rep) were compared to non-repopulating ELSK cells (ELSK_non rep), as well as both repopulating and non-repopulating non-ELSK progenitor cells (Prog)^41^. Heatmaps depict normalized gene expression. Source Data

To decipher why MYCT1 is required for HSC function, we performed single-cell RNA-seq (scRNA-seq) on uncultured CB HSPCs (CD34^+^CD38^–^CD90^+^) and evaluated the correlation of MYCT1 expression and stemness programmes within undifferentiated HLF^+^ HSCs^21,22^ (Extended Data Fig. 4a). HLF^+^ HSCs expressing MYCT1 showed significantly higher expression of several genes associated with HSC identity and function (for example, *MLLT3*, *HIF1A* and *MEIS1*) and lower expression of *CDK6*, a gene associated with ST-HSCs and HSC activation^32^, than HLF^+^MYCT1^–^ and HLF^–^ fractions (Fig. 2a and Supplementary Table 2). To investigate the immediate effects of MYCT1 loss, CB HSPCs were transduced with a *MYCT1* KD vector and resorted (CD34^+^CD38^–^CD90^+^) 72 h after transduction for scRNA-seq. Because MYCT1 transcript levels decrease even in control cultures during this time, *MYCT1* overexpression (OE) was used to rescue MYCT1 levels (Extended Data Fig. 4a). *MYCT1* KD and *MYCT1* OE did not alter the proportion of HLF^+^ cells within sorted HSPCs (Extended Data Fig. 4b–e). Despite their functional defects, *MYCT1* KD HSCs retained even higher expression of some HSC identity genes (for example, *HLF*, *AVP* and *MECOM*) than control HSCs. Nevertheless, expression of many human HSC stemness signature genes^18,33–36^ (for example, *MLLT3*, *HOXA9* and *PROM1*) was downregulated in *MYCT1* KD and upregulated in *MYCT1* OE HLF^+^ HSCs (Extended Data Fig. 2f,g). Functional enrichment analysis of differentially expressed genes linked MYCT1 loss in HLF^+^ HSCs to dysregulation of multiple cellular programmes essential for HSC stemness (Fig. 2b and Supplementary Table 3). The expression of many ETS factors, including the known HSC regulators *ERG* and *ETV6* (ref. ^37^), were reduced with *MYCT1* KD, whereas *MYCT1* OE had the opposite effect (Fig. 2c). MYCT1 loss triggered rapid upregulation of genes involved in mitochondrial respiratory complexes and OXPHOS, which are induced during culture-associated stress and linked to decreased HSC self-renewal ability^24,25^. FACS analysis verified increased mitochondrial membrane potential (TMRE) and mitochondrial reactive oxygen species (MitoSOX) in *MYCT1* KD HSPCs, whereas *MYCT1* OE suppressed them (Fig. 2d–f). MYCT1 loss also dysregulated programmes essential for HSC fate regulation, such as spindle/M phase genes, splicing, ribosomal and proteostasis programmes^26,38–40^, thereby aggravating the effects observed during HSC culture. By contrast, *MYCT1* OE showed profiles more similar to uncultured HSCs (Fig. 2b and Extended Data Fig. 4h–k). The dysregulation of MYCT1 expression and associated programmes in culture was corroborated in an independent human HSPC culture dataset^28^ (Extended Data Figs. 1d and 5a,b). Furthermore, *Myct1* expression and many MYCT1-associated programmes identified in human HSPC culture correlated with functional competence in cultured mouse immunophenotypic HSCs^41^ (Extended Data Fig. 5c–e). Notably, *Myct1* was among 17 genes found in a shRNA screen to be essential for HSC repopulation in mice^42^. These data indicate that the loss or gain of MYCT1 expression induces substantial effects on programmes that regulate human HSC functional competence.

## Restoring MYCT1 improves HSC culture

**Fig. 3:**
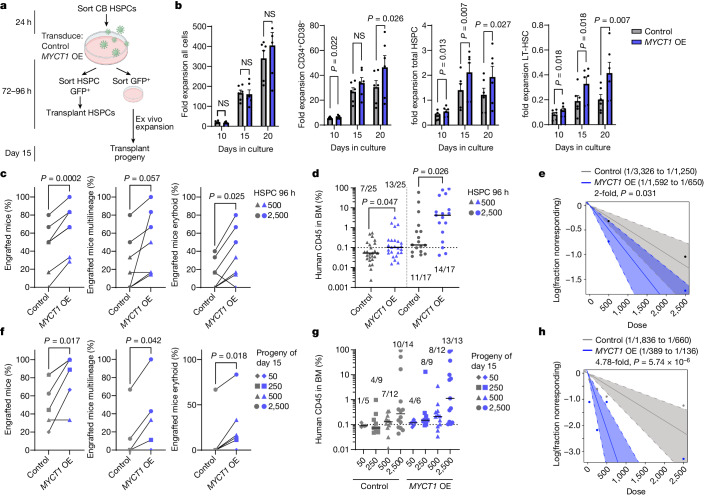
Restoring MYCT1 expression in cultured human HSPCs improves ex vivo expansion and engraftment ability. Experimental outline (**a**) for evaluating the effects of *MYCT1* OE on CB HSPC expansion (**b**) and engraftment ability before (**c**–**e**) and after (**f**–**h**) extended culture. **b**, Fold expansion of control or *MYCT1* OE total cells, CD34^+^CD38^–^ cells, total HSPCs (CD34^+^CD38^–^CD90^+^CD45RA^–^) and immunophenotypic LT-HSCs (CD34^+^CD38^–^CD90^+^CD45RA^–^EPCR^+^ITGA3^+^). *n* = 6 replicates from 3 independent experiments, mean ± s.e.m., ratio-paired two-tailed *t*-test. **c**–**e**, A total of 500 or 2,500 control or *MYCT1* OE HSPCs were sorted (CD34^+^CD38^–^CD90^+^GFP^+^) and transplanted 96 h after transduction into NBSGW mice. **c**, Percentage of mice with human haematopoietic (≥0.1% human CD45^+^), multilineage (myeloid, B lymphoid, and T lymphoid or other) or human erythroid (CD71^+^GlyA^+^) engraftment. Two-tailed paired *t*-test. **d**, Percentage of total human CD45 cells in BM. Median and all individual values are shown, two-tailed Mann–Whitney test. **e**, Estimation of repopulating unit (RU) frequency within transplanted cells after 96 h. **f**–**h**, The progeny of control or *MYCT1* OE HSPCs sorted GFP^+^ 72 h after transduction transplanted after 10 additional days in culture (15 days total). **f**, Percentage of mice with human haematopoietic, multilineage or erythroid engraftment. Two-tailed paired *t*-test. **g**, Percentage of human CD45 cells in the BM for the different doses. Median and individual values are shown. **h**, Estimated RU frequency within transplanted cells at day 15. In **e** and **h**, the frequency of reconstituting units and *P* values were calculated using ELDA^41^. In **c**–**e**, *n* = 25 mice for 500 HSPCs and *n* = 17 mice for 2,500 HSPCs for each group transduced with control and *MYCT1* OE vectors. See Extended Data Fig. 7a and Supplementary Table 4 for mouse numbers and sex of the mice. For **f**–**h**, a total of *n* = 34 mice for each group transduced with control and *MYCT1* OE vectors. See Extended Data Fig. 8a and Supplementary Table 5 for distribution of mice and sex of the mice. For **c**–**h**, engraftment was assessed in BM 12 weeks after transplantation. Schematic in **a** was created with BioRender (https://www.biorender.com). Source Data

**Extended Data Fig. 6:**
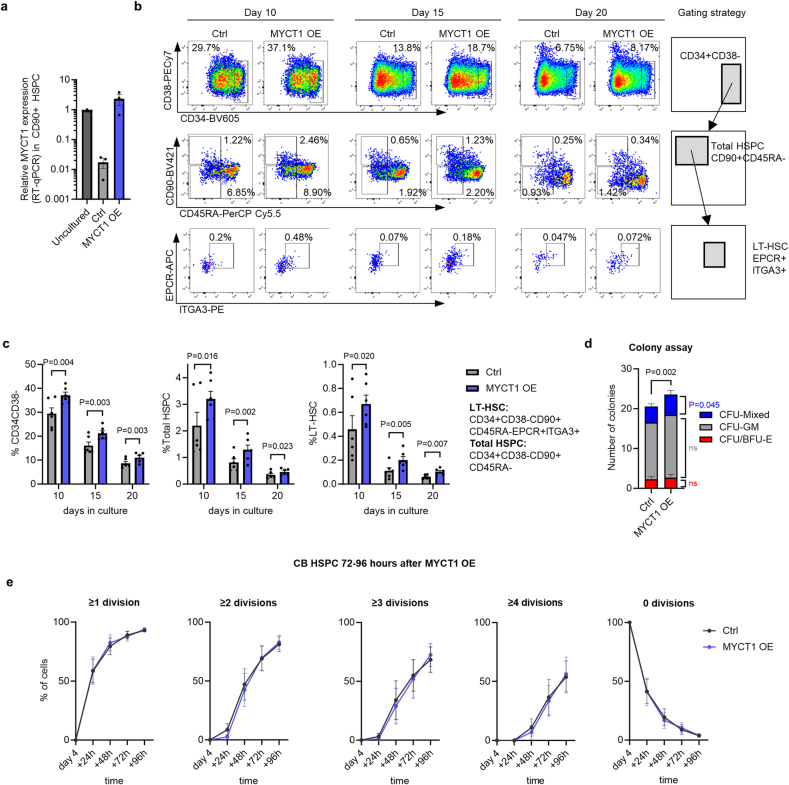
Effects of restoring MYCT1 expression on human HSPCs in culture. **a** Relative MYCT1 expression by RT-qPCR evaluating in sorted control or MYCT1 OE CB HSPCs (CD34^+^CD38^−^CD90^+^GFP^+^) 72 h after transduction. n = 3 independent experiments, mean±s.e.m. **b** Representative FACS plots and gating strategy (**b**), and percentage (**c**) of undifferentiated CD34^+^CD38^−^ cells, HSPCs (CD34^+^CD38^−^CD90^+^CD45RA^−^), and immunophenotypic LT-HSC (CD34^+^CD38^−^CD90^+^CD45RA^−^EPCR^+^ITGA3^+^) expanded from control or MYCT1 OE CB HSPCs after 10, 15 and 20 days in culture. Corresponds to quantifications in Fig. 3b. Percentage of cells in each population within the total cells is indicated in the FACS plots. n = 6 replicates from 3 independent experiments, mean±s.e.m, ratio-paired two-tailed t test. **d** Number and type of colonies formed in enriched methylcellulose (see Methods) after plating equal number of control or MYCT1 OE CB HSPCs (CD34^+^CD38^−^CD90^+^GFP^+^) sorted 72-96 h after transduction. CFU/BFU-E: Colony-forming unit-erythroid or burst-forming unit-erythroid erythroid, CFU-GM: granulocyte and/or macrophage, CFU-Mixed: granulocyte, erythroid, macrophage. n = 4 independent experiments, mean±s.e.m, paired two-tailed t test. **e** Percentage of cells that undergo 1 to 4 divisions, or no divisions observed for every 24 h elapsed since plating. n = 3 independent experiments, mean±s.e.m. Source Data

Our results showed that MYCT1 expression is rapidly lost during HSC culture and *MYCT1* OE HSCs have transcriptional profiles associated with improved HSC functional competency. Therefore, we investigated whether maintaining MYCT1 expression comparable to uncultured HSPCs also improves HSPC function (Fig. 3a). *MYCT1* OE prolonged culture maintenance and increased expansion of cells with the LT-HSC surface phenotype (CD34^+^CD38^–^CD90^+^CD45RA^–^EPCR^+^ITGA3^+^). The total HSPC fraction (CD34^+^CD38^–^CD90^+^CD45RA^–^) and downstream HPC populations (CD34^+^CD38^–^CD90^–^CD45RA^–^ and CD34^+^CD38^–^) were also moderately expanded with *MYCT1* OE (Fig. 3b and Extended Data Fig. 6a–c). Methylcellulose colony assays with *MYCT1* OE HSPCs (CD34^+^CD38^–^CD90^+^) sorted 72–96 h after transduction showed no evidence of differentiation block, but revealed an increase in mixed (granulocyte, erythroid and macrophage) colonies that modestly increased the total colonies. Nevertheless, *MYCT1* OE did not lead to excessive HSPC proliferation (Extended Data Fig. 6d,e). These data suggest that restoring MYCT1 levels during culture improves the expansion of the most undifferentiated human CB HSPCs without disrupting the fate of their downstream progeny.

## Restoring MYCT1 improves HSC engraftment

**Extended Data Fig. 7:**
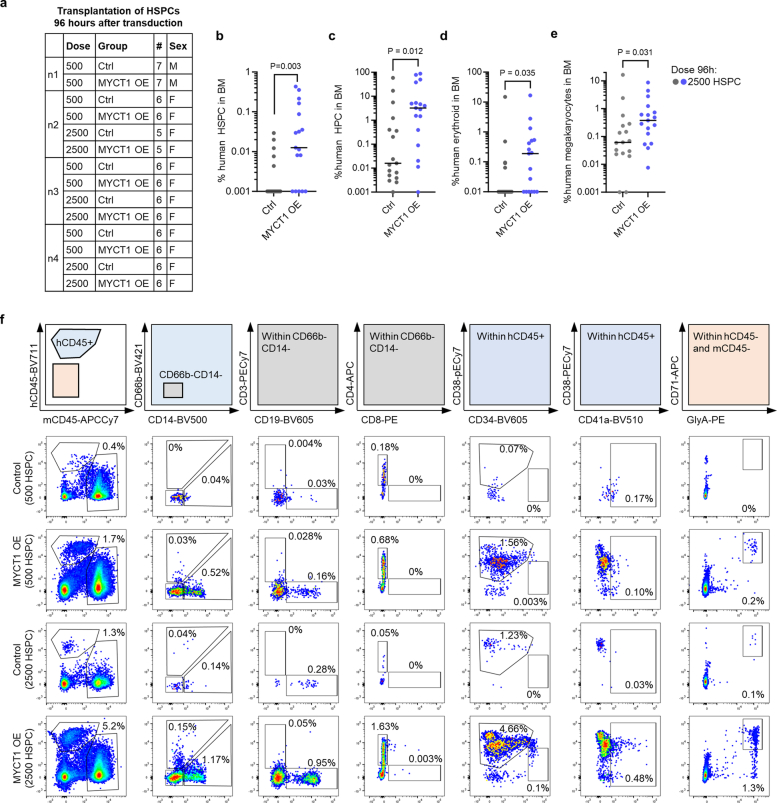
Multilineage differentiation after transplantation of HSPCs with restored MYCT1 expression. **a** Table indicating the number of mice transplanted with control or MYCT1 OE HSPC for each condition per replicate, for the transplantations performed 96 h after transduction. **b-e** Percentage of human undifferentiated HSPC (CD34^+^CD38^−^) (**b**), hematopoietic progenitor cells (HPC, CD38^+^) (**c**), erythroid (CD71^+^GlyA^+^) (**d**), and megakaryocytes (CD41a^+^) (**e**) in BM from NBSGW mice 12 weeks after transplantation with 2500 HSPC. n = 17 mice per group. Median and all individual values are shown, two-tailed Mann–Whitney test. **f** Gating strategy and representative FACS plots showing human myeloid (CD14^+^ or CD66b^+^), B-lymphoid (CD19+), T-lymphoid/other (CD3, CD4 or CD8^+^), undifferentiated HSPC (CD34^+^CD38^−^), HPC (CD38^+^), megakaryocytes (CD41a^+^), and erythroid (GlyA^+^CD71^+^) populations of mice transplanted with 500 or 2500 HSPCs 96 h after transduction. Percentage of cells in each population within the total BM hematopoietic compartment (human and mouse CD45) is indicated in the FACS plots. Source Data

We next evaluated whether the MYCT1-expressing culture-expanded HSPCs also performed better in vivo. We transplanted equal numbers of *MYCT1* OE and control CB HSPCs (500 and 2,500 CD34^+^CD38^–^CD90^+^ cells) into NBSGW mice 96 h after transduction (Fig. 3a). The NBSGW mouse model was chosen as it facilitates human multilineage engraftment (including erythroid and megakaryocytic) without irradiation, thereby avoiding radiation toxicity to the niche^43^. *MYCT1* OE in HSPCs increased the percentage of engrafted mice compared with control transduced HSPCs at 12 weeks after transplantation. These HSPCs also conferred higher engraftment levels of total human haematopoietic cells (CD45^+^), HSPCs (CD34^+^CD38^–^), HPCs (CD38^+^), erythroid cells (CD71^+^GlyA^+^) and megakaryocytes (CD41a^+^) (Fig. 3c,d, Extended Data Fig. 7a–f and Supplementary Table 4). Limiting dilution analysis (LDA)^44^ estimated a twofold improved frequency of engraftable HSCs among the same number of immunophenotypic HSPCs 96 h after transduction (Fig. 3e).

**Extended Data Fig. 8:**
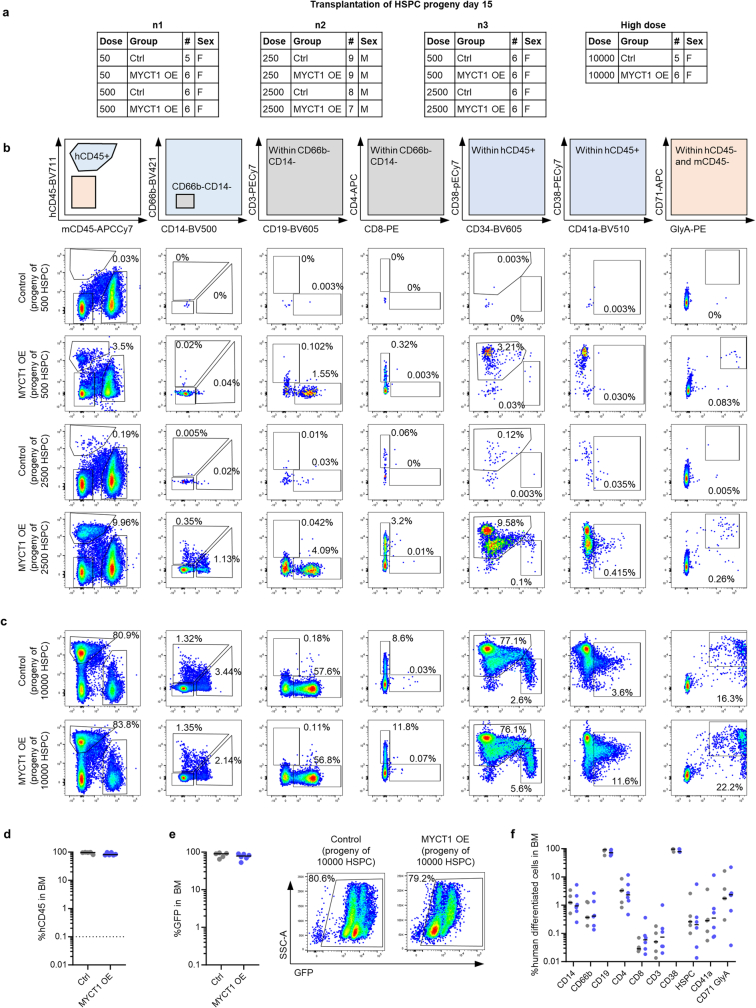
Multilineage differentiation after transplanting day 15 progeny of HSPCs with restored MYCT1 expression. **a** Table indicating the number of mice transplanted with the progeny of control or MYCT1 OE HSPC after 15 days in culture for each condition and replicate. **b,c** Gating strategy and representative FACS plots showing the human myeloid (CD14^+^ or CD66b^+^), B-lymphoid (CD19^+^), T-lymphoid/other (CD3, CD4 or CD^8+^), undifferentiated (CD34^+^CD38^−^), HPC (CD38^+^), megakaryocytes (CD41a^+^), and erythroid (GlyA^+^CD71^+^) populations of mice transplanted with the progeny of 500, 2500 (**b**) or 10000 (high dose) (**c**) HSPCs after 15 days in culture. **d-f** Percentage of total engraftment (hCD45^+^), GFP, and differentiated populations for mice transplanted with the progeny of 10000 HSPCs. n = 5 (control) or n = 6 (MYCT1 OE) mice. Percentage of cells in each population within the total BM hematopoietic compartment (human and mouse CD45) is indicated in the FACS plots. Median and all individual values are shown. Source Data

To determine whether *MYCT1* OE HSPCs cultured over a longer period could sustain improved functionality compared with controls, the progeny of low to intermediate doses (50, 250, 500 and 2,500) of HSPCs sorted for GFP^+^ cells were transplanted after 15 days in culture (Fig. 3a). The frequency of engrafted mice improved with *MYCT1* OE, and even the low doses (250 or 500 cells) of *MYCT1* OE HSPCs generated multilineage and erythroid engraftment in some mice (Fig. 3f,g, Extended Data Fig. 8a,b and Supplementary Table 5). Furthermore, transplantation of the progeny of high doses (10,000), which resulted in consistent, saturating BM engraftment of both control and *MYCT1* OE mice, produced similar multilineage distribution. This result confirmed that sustained MYCT1 expression does not prevent normal differentiation. The expression of GFP was also maintained in vivo, which indicated that MYCT1 expression was sustained (Extended Data Fig. 8c–f). LDA^44^ estimated a 4.8-fold greater increase in the frequency of engraftable HSCs with *MYCT1* OE than with control HSPCs cultured in state-of-the-art conditions with SR1 and UM171 for 15 days (Fig. 3h).

These data indicate that maintaining MYCT1 expression during ex vivo expansion improves the function of human HSPCs both in culture and after transplantation. They also link the reduction in MYCT1 expression to the dysfunction of human HSPCs expanded in conditions involving cytokines and small molecules to support culture of these cells.

## MYCT1 localizes in endosomes in HSCs

**Fig. 4:**
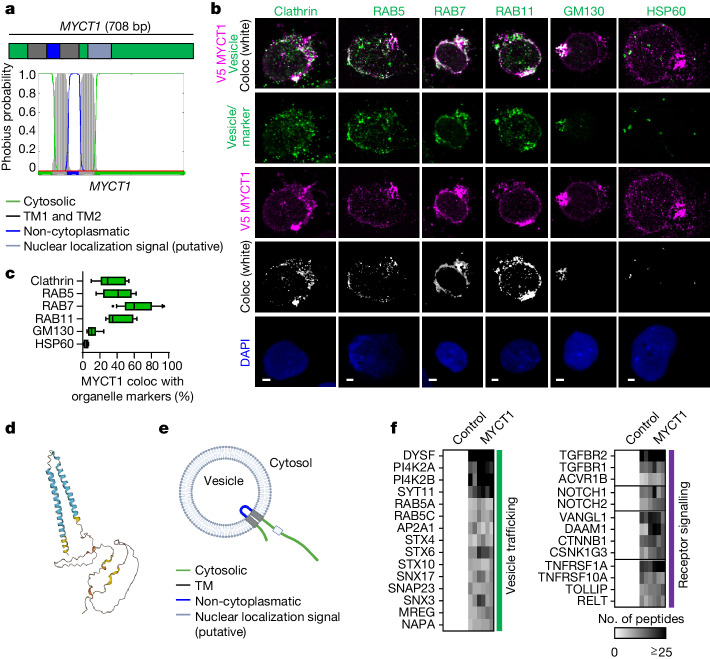
MYCT1 is located in endosomes and interacts with vesicle trafficking and receptor signalling machinery. **a**, The transmembrane topology of MYCT1 was predicted from its amino acid sequence using Phobius^48^ and is depicted as a schematic and histogram of probability. **b**,**c**, High-resolution Airyscan immunofluorescence images (**b**) and quantification (**c**) of the localization of overexpressed MYCT1–V5 in human CB HSPCs after 5 days of culture. MYCT1–V5 was visualized by staining for V5, and colocalization with endosomal markers (clathrin, RAB5, RAB7 and RAB11), Golgi marker (GM130) and mitochondrial marker HSP60 was evaluated. DAPI indicates nuclei. Colocalization channel (Coloc) shows areas positive for V5 and each marker. Scale bars, 3 µm (RAB5) or 2 µm (other columns). Analysis was performed using Imaris (v.9.7.2) software on *n* = 8 (clathrin), *n* = 5 (RAB5), *n* = 13 (RAB7), *n* = 6 (RAB11), *n* = 4 (HSP60) and *n* = 6 (GM130) images per condition from 2 independent experiments. Box plots depict median and 10–90th percentile, and data above and below are shown. **d**, Prediction of the MYCT1 3D structure reproduced from AlphaFold (https://alphafold.com/entry/Q8N699)^49,50^. **e**, Model of the proposed MYCT1 structure, localization and topology. **f**, Heatmap of selected MYCT1 interactors detected by IP of the V5 tag in control KG1 cells and in MYCT1–V5 OE KG1 cells followed by high-sensitivity MS. Number of detected peptides for each protein are shown. *n* = 3 independent experiments, technical duplicates for each experiment are shown. Schematic in **e** was created with BioRender (https://www.biorender.com). Source Data

**Extended Data Fig. 9:**
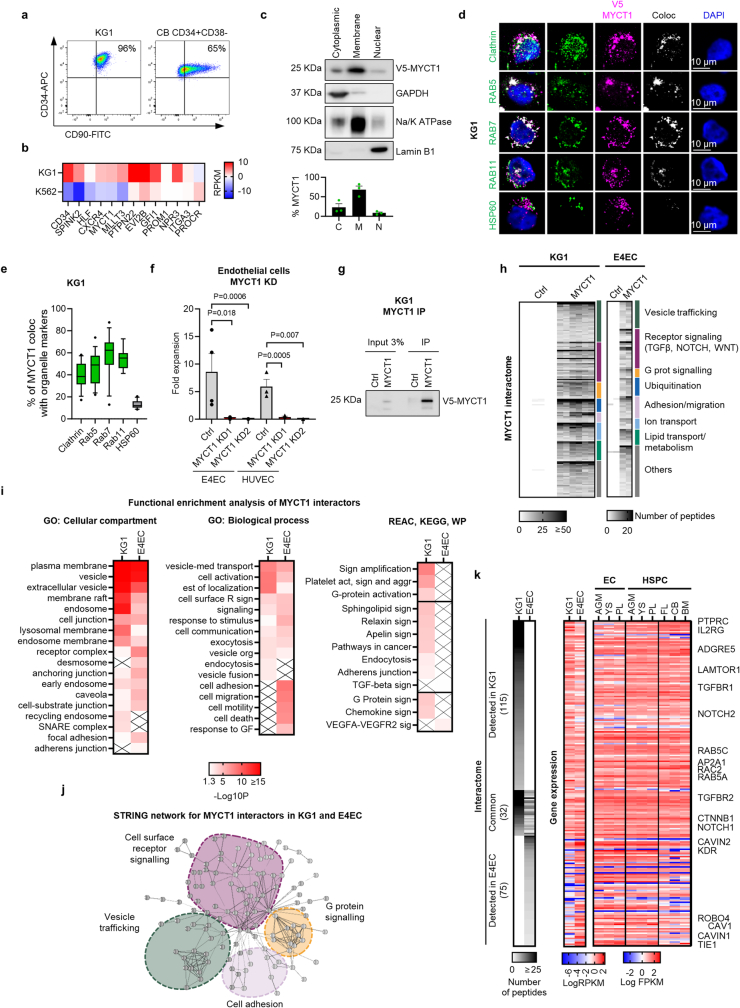
Dissecting the MYCT1 interactome in KG1 hematopoietic cells and endothelial cells. **a-f** KG1 cells and E4EC as models for studying MYCT1 function in HSC. **a** FACS analysis of HSC surface markers CD34 and CD90 in KG1 cells and human CB HSPCs. n = 1 experiment. **b** Gene expression (RNAseq) for HSC genes in KG1 and K562 cells from the Cancer Cell Line Encyclopaedia^76^. **c** Western blot of MYCT1 in subcellular fractions of KG1 cells overexpressing (OE) MYCT1-V5. Representative image and quantification from n = 3 independent experiments, mean±s.e.m. Lamin B1, GAPDH, and Na/K ATPase are fraction controls. For gel source data, see Supplementary Fig. 3. **d,e** Confocal immunofluorescence and quantification of MYCT1-V5 and co-localization with endosomal (Clathrin, Rab5, Rab7, Rab11) and mitochondrial (HSP60) markers in KG1 cells. DAPI indicates nuclei. Colocalization channel (coloc) shows areas positive for V5 and each marker. Scale bars = 10 μm. Representative images of n = 3 independent experiments. Colocalization was assessed with Imaris v9.7.2 software on n = 16 (clathrin), n = 17 (Rab5), n = 12 (Rab7), n = 9 (Rab11), n = 11 (HSP60) images per condition. Box plots depict median and 10-90 percentile, data above and below are shown. **f** Fold expansion of E4EC and non-immortalized HUVEC cells 15 days after transduction with control or MYCT1 KD vectors. n = 4 (E4EC) or n = 3 (HUVEC) independent experiments, mean±s.e.m., two-tailed ratio paired t-test. **g-j** Dissecting the MYCT1 interactome. **g** Western blot for immunoprecipitation of V5-tagged MYCT1 in control or MYCT1 OE KG1 cells. Representative of n = 2 experiments. For gel source data, see Supplementary Fig. 4. **h** Heatmap of MYCT1 interactors detected by immunoprecipitation of V5 tag in control or MYCT1 OE KG1 and E4EC cells. Scale depicts the number of detected peptides for each protein. Colours indicate protein categories. n = 3 (KG1) or n = 1 (E4EC) independent experiments, technical duplicates from each experiment are shown. **i** Heatmaps of -LogP values of GO, Reactome (REAC), KEGG, and WikiPathway (WP) terms for MYCT1 interactome in KG1 and E4EC. Functional enrichment analysis was performed using g:Profiler^69^ with g:SCS multiple testing correction method applying significance threshold of 0.05. **j** Interaction network depicting high confidence functional and physical associations of MYCT1 interactors in KG1 and E4EC generated using STRING^51^ v11.5. **k** Heatmaps showing MYCT1 interactors in KG1 and E4EC by number of peptides, and their average expression from RNAseq in KG1, E4EC (in RPKM, reads per kilobase of transcript per million reads mapped) and sorted ECs and HSPCs from embryonic tissues (5-6 week AGM, aorta-gonad-mesonephros, YS, yolk sac, PL, placenta), second trimester FL, CB and adult bone marrow (ABM) (in FPKM, fragments per kilobase of transcript per million reads mapped). Source Data

The structure and molecular function of MYCT1 have been poorly defined, although some reports suggest that MYCT1 may act as a nuclear factor or a membrane protein in different cell types^45–47^. Based on its amino acid sequence, MYCT1 is predicted to have two transmembrane (TM) domains and a putative nuclear localization signal. Topology prediction^48^ predicted that the amino-terminal and carboxy-terminal ends are cytoplasmic, with a short non-cytoplasmatic region between the two TM domains (Fig. 4a). Evaluation of the localization of V5-tagged MYCT1 in KG1 cells (an AML cell line with human HSPC-like surface phenotype and gene expression programs) revealed that MYCT1 is enriched in the membrane fraction (Extended Data Fig. 9a–c). High-resolution imaging of V5-tagged MYCT1 and endosomal markers in human CB HSPCs revealed that MYCT1 localized in vesicles. MYCT1 also colocalized with endosomal proteins, including clathrin (responsible for the initial steps of endocytosis), RAB5 (early endosomes), RAB7 (late endosomes) and RAB11 (recycling endosomes). A minor fraction of the MYCT1 signal was observed in the Golgi apparatus (GM130), but no colocalization was found with the mitochondrial marker HSP60, which implied that the effects of *MYCT1* KD and OE on mitochondrial activity are indirect (Fig. 4b,c). Comparable localization of MYCT1 was observed in KG1 cells (Extended Data Fig. 9d,e). The colocalization with endosomal markers, together with the topology prediction and the 3D structure predicted with AlphaFold^49,50^, suggest that in HSCs and HSC-like cells, MYCT1 acts primarily in endosomes and localizes at the membrane of endosomes through the two TM domains, with a short intraendosomal loop (Fig. 4d,e).

## MYCT1 interacts with endosome components

To understand the molecular function of MYCT1, we performed immunoprecipitation coupled with high-sensitivity mass spectrometry (IP–MS) of V5-tagged MYCT1 in haematopoietic cells (KG1) and in E4-immortalized human umbilical vein endothelial cells (HUVECs) (E4EC)^13^. E4EC cells were chosen as an additional discovery model, as they depend on MYCT1 expression for culture expansion, similar to non-immortalized HUVECs (Extended Data Fig. 9a–g). MYCT1 interacted with proteins involved in vesicle trafficking and endosomes (for example, the early endosomal proteins RAB5A and RAB5C and the clathrin adaptor AP2A1). It also interacted with factors involved in cell surface receptor signalling and signal amplification (for example, TGFBR1, TGFBR2, NOTCH1, NOTCH2 and CTNNB1), G protein signalling and cell adhesion. MYCT1-interacting proteins identified in haematopoietic cells (KG1) and ECs (E4EC) belonged to the same families and share functional and/or physical networks (STRING^51^), although many interacting proteins were cell-type-specific (Fig. 4f, Extended Data Fig. 9h–j and Supplementary Table 6a–d). These findings raise the possibility that MYCT1 may act as an endosomal adaptor protein^52^.

To correlate MYCT1 interactors identified in KG1 and E4EC cell lines with primary human haematopoietic cells, we compared the MYCT1 interactome with RNA-seq data of MYCT1-expressing human HSPCs and their precursors from intraembryonic and extraembryonic haemogenic tissues throughout ontogeny (see also Fig. 1a). The interactome genes from KG1 cells were typically expressed most strongly in second trimester FL, CB and adult BM HSPCs. By contrast, the MYCT1 interactors detected only in E4EC cells were more highly expressed in ECs and newly emerged HSPCs in first trimester haematopoietic tissues (5–6-week AGM, yolk sac and placenta) (Extended Data Fig. 9k and Supplementary Table 6e). These data confirm that MYCT1 interacts in endosomes with vesicle trafficking and signalling proteins that are expressed at different stages of human HSC development and ECs.

## MYCT1 controls endocytosis in human HSCs

**Fig. 5:**
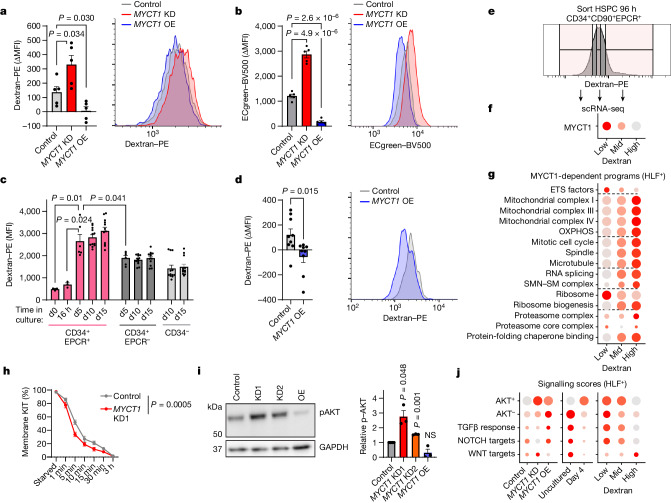
MYCT1 controls endocytosis and environmental sensing in human HSPCs. **a**–**d**, Evaluating the effects of *MYCT1* KD and OE on endocytosis. **a**,**b**, Quantification (left) and representative FACS histogram (right) of low-molecular-weight (10 kDa) fluorescent dextran (**a**) or ECgreen (**b**) internalized over 30 min in control, *MYCT1* KD or OE CB HSPCs 72 h after transduction. *n* = 5 replicates from 3 independent experiments, mean ± s.e.m., two-tailed *t*-test. **c**, Quantification of internalized fluorescent dextran in CB HSPC and HPC subsets over time in culture. *n* = 4 (d0), *n* = 6 (d5), *n* = 12 (d10 and d15) replicates from 4 independent experiments and *n* = 3 from 3 independent experiments (16 h), mean ± s.e.m., two-tailed Mann–Whitney test. **d**, Quantification (left) and representative histogram (right) of internalized fluorescent dextran in control or *MYCT1* OE CB HSPCs (CD34^+^CD90^+^) cultured for 10–12 days. *n* = 9 from 4 experiments, mean ± s.e.m., two-tailed *t*-test. **e**–**g**, Evaluating CB HSPCs with different endocytosis levels. **e**, Experimental outline for scRNA-seq of CB HSPCs (CD34^+^CD38^–^CD90^+^EPCR^+^) with low, medium or high levels of endocytosis after 4 days in culture. **f**,**g**, Dot plots depicting MYCT1 expression (**f**) or the scores for MYCT1-associated functional categories (Fig. 2) (**g**) in HLF^+^ HSCs. **h**, KIT internalization in response to SCF stimulation by FACS in control or *MYCT1* KD CB HSPCs (CD34^+^CD38^–^EPCR^+^). *n* = 3 experiments, mean ± s.e.m., two-way analysis of variance. **i**, Western blot (left) and quantification (right) of phospho-AKT (pAKT) in control, *MYCT1* KD or OE CB HSPCs 72 h after transduction. *n* = 3 experiments, mean ± s.e.m., two-tailed paired *t*-test. GAPDH is a sample processing control. For gel source data, see Supplementary Fig. 1. **j**, Dot plots for signalling scores (scRNA-seq) in control, *MYCT1* KD or OE cells 72 h after transduction, uncultured and day 4 cultured CB HLF^+^ HSCs, and HSCs with low, medium or high dextran internalization. AKT^+^ and AKT^–^ indicate positive regulators and negative regulators, respectively, of AKT signalling. For **a**, **b** and **d**, ΔMFI is between the GFP^+^ and GFP^–^ cells within the same sample. Source Data

Given that MYCT1 localizes in endosomal membrane and interacts with endosomal trafficking proteins, we asked whether MYCT1 regulates endocytosis in human HSCs. The endocytosis rate was quantified by flow cytometry by measuring the internalization of low-molecular-weight (10 kDa) fluorescent dextran or the endocytosis detector ECgreen in cultured human CB HSPCs after 30 min of exposure. Both analyses showed increased endocytosis after *MYCT1* KD, whereas *MYCT1* OE showed a decrease (Fig. 5a,b). Monitoring endocytosis in CB HSPCs during culture showed increased endocytosis mainly in the highly purified HSPC subset (CD34^+^EPCR^+^). Notably, restoring MYCT1 expression was sufficient to suppress the excessive culture-induced endocytosis in HSPCs even after 10–12 days (Fig. 5c,d). These data show that MYCT1 is crucial for controlling the rate of endocytosis in human HSCs.

## Low endocytosis preserves HSC stemness

**Extended Data Fig. 10:**
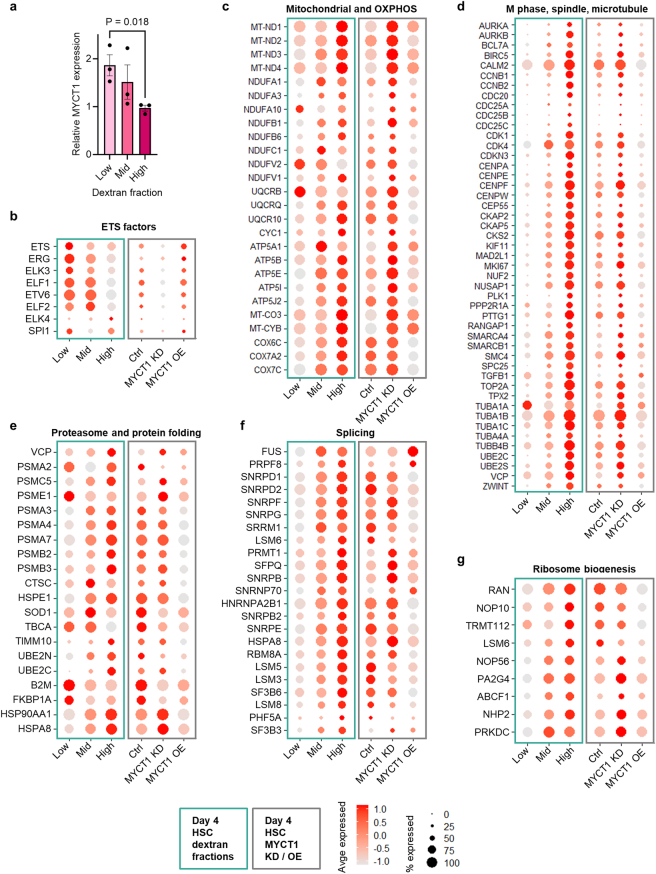
Expression of MYCT1 and MYCT1 associated “stemness” programs in HSCs with low, medium or high endocytosis. **a** Relative expression of MYCT1 by RT-qPCR in highly purified 4-day cultured HSPCs (CD34^+^CD38^−^CD90^+^EPCR^+^) with low, medium or high internalization of fluorescent dextran (10 KDa). n = 3 independent experiments, mean±s.e.m. two-tailed t-test. **b-g** Single cell RNAseq dot plots depicting gene expression for the programmes that were found to be governed by MYCT1. HLF^+^ HSC cultured for 4 days and sorted for low, medium, or high internalization of fluorescent dextran (green box) are compared to control, MYCT1 KD and MYCT1 OE HLF^+^ (grey box) (From Fig. 2 and Extended Data Fig. 4). n = 1 experiment. The number of cells sequenced per fraction are shown in Supplementary Table 7. Source Data

We next asked whether different levels of endocytosis in cultured HSPCs correlate with MYCT1 expression and differences in stemness programmes. We fractionated the highly purified CB HSPCs (CD34^+^CD38^+^CD90^+^EPCR^+^) based on dextran internalization levels after 4 days in culture, and performed scRNA-seq for the subfractions (Fig. 5e). Notably, HSPCs with the lowest internalization of dextran identified HLF^+^ HSCs with the highest MYCT1 expression. Moreover, when compared with HSPCs with medium and high levels of endocytosis, the low endocytosis fraction showed scRNA-seq expression patterns consistent with preservation of stemness properties associated with MYCT1, including maintenance of ETS factors and lower mitochondrial activity (Figs. 2b and 5f,g, Extended Data Fig. 10a–g and Supplementary Table 7). These analyses highlight the importance of MYCT1-mediated endocytosis in governing a broad range of molecular programmes and functions related to HSC stemness.

## MYCT1 controls HSC environmental sensing

**Extended Data Fig. 11:**
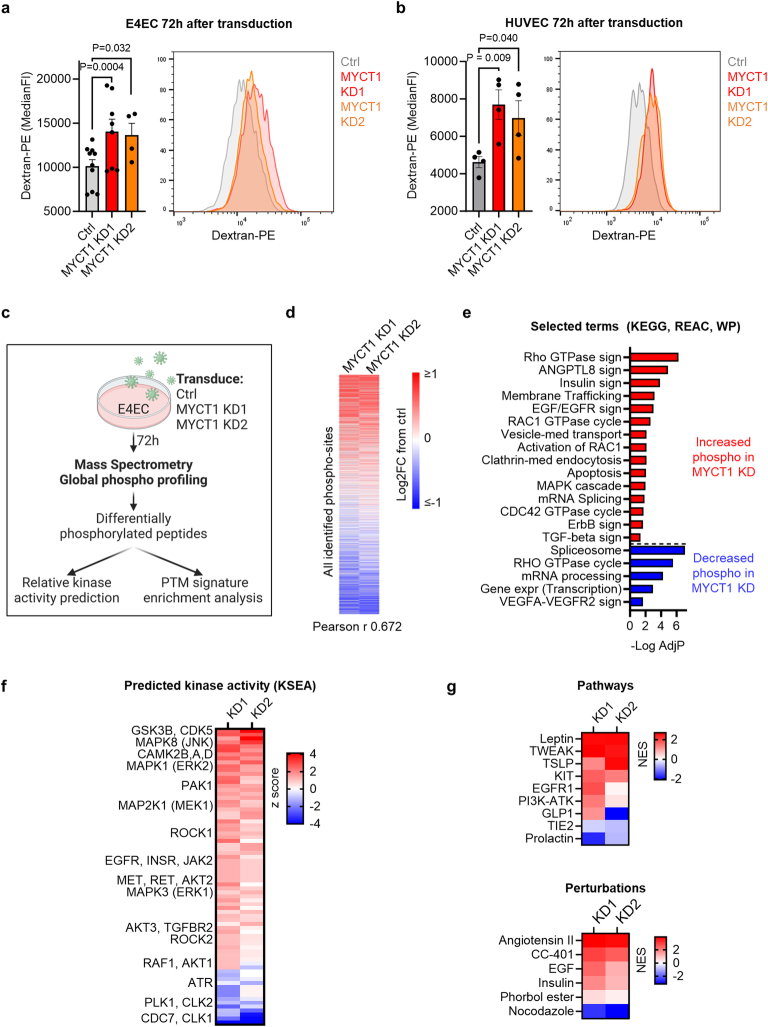
Endothelial cells as a discovery model for evaluating MYCT1 moderated signalling responses. **a,b** Evaluating the effects of MYCT1 KD on endocytosis. Quantification and representative FACS histogram of fluorescent low molecular weight (10 KDa) dextran internalized over 30 min in control or MYCT1 KD E4ECs (**a**) or non-immortalized HUVECs (**b**) 72 h after transduction. For E4EC n = 10 (ctrl), n = 8 (KD1) and n = 4 (KD2) from 3 independent experiments, for HUVEC n = 4 replicates from 3 independent experiments, mean±s.e.m, two-tailed paired t-test. **c-g** Phosphoproteomic analysis of the effects of MYCT1 KD in E4EC 72 h after transduction from n = 1 experiment in technical duplicates. **c** Experimental outline. **d** Heatmap depicting the Log2FC for all identified phosphorylated protein sites. Pearson correlation between the two different MYCT1 KD vectors calculated using Excel is included. **e** Functional enrichment analysis of the differentially phosphorylated proteins. LogP values for selected KEGG, Reactome (REAC) and WikiPathway (WP) terms for the proteins with increased (red) or decreased (blue) phosphorylation after MYCT1 KD compared to control. Performed using g:Profiler^69^ with g:SCS multiple testing correction method applying significance threshold of 0.05. **f** Predicted relative kinase activity (KSEA^59^) in MYCT1 KD compared to control. **g** Predicted pathways and perturbations enriched after MYCT1 KD determined by PTM-SEA^56^. Schematic in **c** was created with BioRender (https://www.biorender.com). Source Data

Endocytosis is a pivotal regulatory step in extracellular signalling that can determine the length, strength and quality of cell signalling from numerous receptors, and must be tightly controlled to maintain adequate responses to external signals^53,54^. To define whether MYCT1 loss dysregulates signalling pathways, we used E4EC cells as a discovery model and performed MS-based global phosphoproteomic profiling in control and *MYCT1* KD E4EC cells. Notably, E4EC cells showed increased endocytosis in response to *MYCT1* KD, similar to human CB HSPCs and non-immortalized HUVECs (Fig. 5a,b and Extended Data Fig. 11a,b). *MYCT1* KD led to global changes in the phosphoproteome of ECs, with increased phosphorylation of proteins involved in EGFR, insulin and Rho-GTPase signalling, as well as proteins involved in membrane trafficking and clathrin-mediated endocytosis (Extended Data Fig. 11c–e and Supplementary Table 8).

Predictions of relative kinase activity (KSEA)^55^ and signature enrichment analysis (PTM-SEA)^56^ revealed signalling hyperactivation in response to MYCT1 loss. *MYCT1* KD E4EC cells showed increased predicted activity of receptor kinases, such as EGFR, INSR or KIT, the downstream signal transduction pathways (PI3K–AKT, ERK1 and ERK2), as well as endocytosis-related kinases (CDK5 and PAK1). By contrast, kinases and pathways involved in cell cycle progression and EC survival (CDC7 and PLK1 kinases, TIE2 and prolactin pathways) were suppressed after MYCT1 loss. Notably, *MYCT1* KD E4EC cells showed enriched phosphoproteomic signatures that have been observed in response to EGF and insulin treatment, although both control and KD cells were cultured in equal concentrations of EGF and IGF1 (Extended Data Fig. 11f,g). These data suggest that MYCT1 loss can trigger broad hyperactivation of receptor-mediated kinase signalling pathways and dysregulation of other signalling networks crucial for proliferation and survival, and may lead to increased sensitivity to the signals in the culture environment.

**Extended Data Fig. 12:**
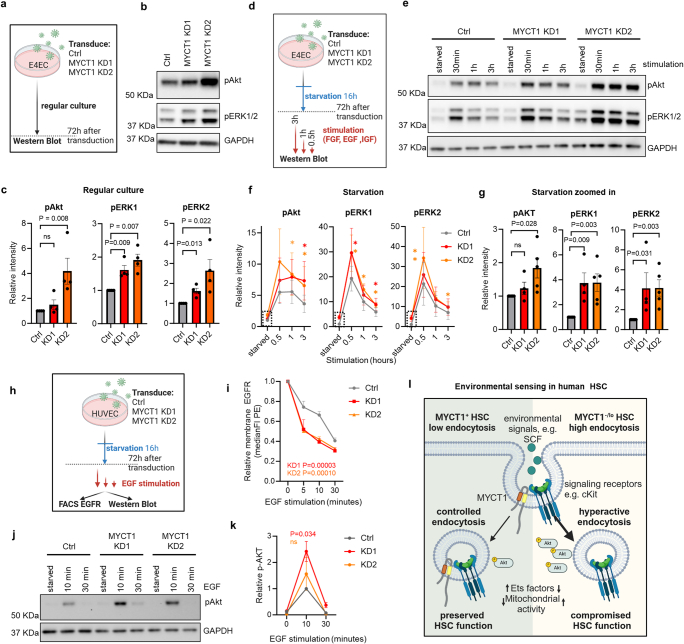
Evaluation of MYCT1 moderated signalling responses in endothelial cell models. **a-c** Quantifying basal signalling responses by western blot in control and MYCT1 KD E4EC cultured with continuous presence of serum and cytokines. **a** Experimental outline. **b,c** Representative western blot (**b**) and quantification (**c**) of phospho-AKT and phospho-ERK in control and MYCT1 KD E4ECs. n = 3 independent experiments, mean±s.e.m, two-tailed ratio-paired t-test. **d-g** Determining the effect of MYCT1 KD on the signalling responses to cytokine stimulation by western blot in control or MYCT1 KD E4EC. **d** Experimental outline. **e-g** Representative western blot (**e**) and quantification of phospho-AKT and phospho-ERK in control and MYCT1 KD E4ECs after overnight starvation and subsequent stimulation with complete media (containing serum, FGF, EGF and IGF) for 30 min, 1 and 3 h (**f**). The quantifications under starvation conditions are also shown zoomed in (**g**). n = 4 (KD1) or n = 5 (KD2) independent experiments, mean±s.e.m, two-tailed ratio-paired t-test. *P* values in **m** from left to right for KD1: 0.0152 (pAKT), 0.0491, 0.0288 (pERK1); for KD2: 0.0211, 0.0359 (pAKT), 0.0442, 0.0045, 0.00515 (pERK1), 0.0095, 0.0267 (pERK2). **h-k** Determining the effect of MYCT1 KD on EGFR internalization and downstream signalling in response to EGF stimulation in non-immortalized HUVECs 72 h after transduction. **h** Experimental outline. **i** Quantification of relative membrane EGFR by FACS in starvation or in response to EGF stimulation for 5, 10, and 30 min. n = 3 independent experiments, mean±s.e.m, two-way ANOVA. **j,k** Representative western blot (**j**) and quantification (**k**) of phospho-AKT in control and MYCT1 KD HUVEC after overnight starvation and subsequent stimulation with EGF for 10 and 30 min. n = 4 independent experiments, mean±s.e.m, two-tailed paired t-test. **l** Model figure of MYCT1 regulated environmental sensing in HSC. For gel source data, see Supplementary Fig. 5. GAPDH is a loading control for AKT in **b** and **j**, and a sample processing control for pERK1/2 in **b** and all antibodies in **e**. Schematics in **a,d,h,l** were created with BioRender (https://www.biorender.com). Source Data

In accordance with the phosphoproteomic signatures, *MYCT1* KD E4EC cells cultured in the continuous presence of serum and cytokines showed a constant basal hyperactivation of AKT and ERK, at which multiple receptor signalling pathways converge (Extended Data Fig. 12a–c). This hyperactive signalling was due to an increase and prolongation of the normal responses to cytokine stimulation. That is, increased phosphorylation of AKT and ERK were observed in *MYCT1* KD cells after stimulation with the standard cytokines present in the culture medium (serum, EGF, IGF1 and FGF). Even in starvation conditions, *MYCT1* KD led to greater AKT and ERK activation (Extended Data Fig. 12d–g). We sought to understand how MYCT1 loss leads to hypersensitivity to environmental signals and to eliminate potential artefacts produced by EC immortalization. We monitored internalization of EGFR in response to EGF stimulation, which is known to occur through endocytosis^53,54^, and the downstream signalling response in non-immortalized HUVECs. EGFR internalization occurred faster in *MYCT1* KD cells than in control cells, and led to hyperactivation of phospho-AKT (Extended Data Fig. 12h–k). This result links increased receptor internalization to overactive downstream signalling responses.

Confirming our findings from EC discovery models, *MYCT1* KD in human CB HSPCs also resulted in increased internalization of the crucial HSC receptor KIT in response to stimulation with its ligand SCF, which is also known to occur through endocytosis and signal through AKT^57^ (Fig. 5h). When cultured in standard HSC-supportive conditions (SCF, FLT3-L, TPO with UM171 and SR1), *MYCT1* KD HSPCs showed AKT hyperactivation, which is detrimental for HSC function when induced above tolerable levels^58,59^ (Fig. 5i). Because endocytosis controls signalling from multiple receptors in different manners, we interrogated scRNA-seq data for responses to different signalling pathways. Module score analysis also suggested broad dysregulation of signalling pathways in *MYCT1* KD CB HSPCs, including hyperactive AKT signalling, decreased responses to TGFβ and NOTCH signalling and increased response to WNT signalling, all known to be regulated by endocytosis^54^. Similar signalling aberrations were observed in high endocytosis HSCs, whereas rescue by *MYCT1* OE and low endocytosis HSCs balanced these signalling responses (Fig. 5j and Supplementary Tables 3 and 7). These data imply that MYCT1 controls environmental sensing in human HSCs to fine-tune signalling responses and maintain HSC self-renewal and engraftment ability.

## Discussion

Here we discovered a crucial role for HSC and the endothelial gene *MYCT1* in preserving human HSC self-renewal and engraftment ability during culture, which is due to its function in controlling endocytosis and environmental sensing (Extended Data Fig. 12l). Loss of MYCT1 expression during human HSC culture led to hyperactivation of endocytosis and cytokine signalling and complete abrogation of HSC function. Maintaining MYCT1 expression during HSC culture was sufficient to dampen culture-induced excessive endocytosis and support the expansion of immunophenotypic human HSCs that improved transplantation outcomes in the immunodeficient NBSGW mouse model.

Endocytosis is classically viewed as the initial step in the endolysosomal pathway that leads to signalling termination through degradation of membrane receptors, but it can also act as pro-signalling platform^53,54^. Our findings that hyperactivation of endocytosis in human HSCs following MYCT1 loss is commonly coupled with excessive rather than dampened receptor-mediated signalling highlight the pro-signalling role of endocytosis in HSC biology, and suggest that HSCs rely on low levels of endocytosis to maintain signalling responses within tolerable thresholds. Endocytosis not only controls signalling from multiple signalling receptors but also has a wide impact on cellular homeostasis. Endocytosis controls metabolism through the membrane availability of nutrient receptors and ion channels (for example, cholesterol and iron uptake), migration, adhesion, proliferation, polarity and immune responses, among other cell processes^60^. Therefore, the far-reaching effects triggered by MYCT1 loss can be explained by the dysregulation of endocytosis that alters the responses to a myriad of environmental signals. These mechanisms together with hyperactive AKT signalling may be contributing to the increased mitochondrial activity, higher OXPHOS and impaired HSC self-renewal associated with MYCT1 loss and HSC culture more broadly^58^.

Despite endocytosis being an elemental process in all cell types, MYCT1 expression is selectively enriched in the most undifferentiated HSCs. Recent reports showed that endocytosis balances signalling cues to maintain pluripotency in embryonic stem cells^61,62^, controls HSC emergence from haemogenic endothelium in zebrafish^63^ and BM homing in mice after transplantation^64^. We propose that owing to the intrinsic need of HSCs to switch between fates in a controlled, and in part reversible fashion, HSCs may require stricter control of endocytosis and environmental sensing mechanisms to maintain stemness than in more differentiated cells. Indeed, we observed that HSCs with lower endocytosis levels show improved preservation of stemness programmes compared with endocytosis-high HSCs with equivalent HLF expression and immunophenotype. HSCs also require more precise control of mitochondrial activity^24,25^, proteostasis^26,27^ and lysosomal activity^28^ than more differentiated cells and are less tolerant to imbalances that happen during stress, such as culture. Notably, many of these programmes were influenced by MYCT1. Our discovery draws the attention to the essential cellular activities that HSCs must fine-tune to maintain their specific functional properties during ex vivo expansion, and uncovers MYCT1-controlled endocytosis as a crucial regulatory node for these processes.

Despite the recent advances in understanding cellular and molecular mechanisms that are vital for maintaining HSC identity and self-renewal, the ability to control these processes ex vivo to improve HSC-based therapeutic applications has been a long-standing challenge. Our work highlights the importance of not just providing the crucial extrinsic signals in the culture environment but also maintaining low endocytosis and preserving the specific regulatory machinery in HSCs that can properly process these signals. Thus, improving the niche must be coupled with strategies aimed at maintaining the machinery responsible for environmental sensing, including MYCT1. So far, it is unknown why most reported culture conditions induce substantial MYCT1 downregulation and epigenetic silencing of the *MYCT1* locus. Identifying the upstream regulators and regulatory elements that govern MYCT1 expression may provide key insights into the mechanisms that underlie MYCT1 silencing and culture-induced stress. Future studies will be needed to identify the best strategies for maintaining MYCT1 expression and controlled endocytosis in cultured human HSPCs and to validate the beneficial effects of maintaining MYCT1 expression in other in vivo models that support long-term human HSC engraftment. Our work nominates sustained MYCT1 expression and fine-tuned endocytosis as new hallmarks of human HSC quality control after ex vivo expansion.

## Methods

### Reagents

See Supplementary Table 9.

### Human HSPC collection and isolation

First trimester haematopoietic tissues and second trimester FLs (14–18 developmental weeks) were de-identified, discarded material obtained from elective terminations of pregnancy after informed consent. First trimester tissues (5–6 weeks) include the AGM region, the placenta and the yolk sac. CB units were obtained at birth following informed consent and de-identified after collection. Adult BM aspirates were purchased from Allcells.

AGM, placenta and yolk sac were washed in Dulbecco’s phosphate buffered saline (DPBS; Gibco, 14190250) and placed in sterile DPBS with 5% FBS (FB-01, Omega Scientific), 1% penicillin–streptomycin (Thermo Fisher Scientific, 15140-122) and 2.5 µg ml^–1^ amphotericin B (Fisher BioReagents, BP2645-50) and processed for sorting within 48 h. Tissue samples were digested in 2.5 U dispase (Thermo Fisher Scientific, 17105-041), 90 mg collagenase A (Worthington, LS004176) and 0.075 mg DNase I (Sigma-Aldrich, D4513) per ml in DPBS containing 10% FBS for 20–45 min at 37 °C. Cells were disaggregated by pipetting and filtered through a 70 µm cell strainer. FLs were collected into DPBS with 5% FBS and mechanically dissociated using scalpels and syringes before proceeding with enzymatic dissociation described above. CB was diluted 1:2 with DPBS containing 2% FBS, 1 mM EDTA (Invitrogen, AM9260G) and 4.2 U ml^–1^ DNAse I before proceeding to the enrichment of mononuclear cells.

For FL, CB and adult BM, mononuclear cells were enriched on SepMate-50 tubes using Lymphoprep (StemCell Technologies, 85450 and 07861) layer following the manufacturer’s protocol and filtered through a 70 µm cell strainer. CD34^+^ cells were enriched using human CD34 MicroBead Kit UltraPure (Miltenyi Biotec 130-100-453). Cells were viably frozen or sorted for experiments directly.

### Ethics statement

First trimester tissue samples were obtained from the University of Tubingen and delivered to the University of California Los Angeles (UCLA) within 48 h after the procedure. The Ethics Committee at the Medical Faculty of the Eberhard Karls University Tübingen and at the University Hospital in Tübingen approved the use of human embryo tissues from elective terminations for HSC research (number 290/2016BO1). Second trimester FL tissues were obtained from elective terminations performed at UCLA or at Family Planning Associates and provided to the UCLA CFAR Cell and Gene Therapy core for distribution to UCLA investigators. All human fetal tissue used were discarded material from elective terminations that were obtained following informed consent. The donated human fetal tissues were anonymized and did not carry any personal identifiers. In all cases, the decision to terminate the pregnancy occurred before the decision to donate tissue. No payments were made to donors and the donors knowingly and willingly consented to provide research materials without restrictions for research and for use without identifiers. CB units were obtained from Cedars Sinai Medical Center following informed consent and de-identified following collection. The UCLA Institutional Review Board (IRB) determined that the provision of anonymized fetal material and CB for research does not constitute human research per the US Federal regulations because the tissues are anonymized, (that is, provided without any direct or indirect identifiers that could be linked back to a living individual). As a result, investigators using such material are not engaged in research subject to IRB oversight. All donors gave informed consent in compliance with US Public Health Service Act, Sections 498A and 498B for the use of fetal material in research. All human tissue materials were treated as Biosafety level 2 and approved by UCLA Institutional Biosafety Committee (BUA-2016-142-001, BUA-2019-186-001).

### Cell lines

Immortalized HUVECs (E4EC)^13^ were obtained from S. Rafii’s laboratory and were cultured on gelatin-coated flasks with Medium 199 (GE Life Sciences, sh30253.01) containing 20% FBS (FB-01, Omega Scientific), 1% penicillin–streptomycin, 1% l-glutamine, 10 mM HEPES (Thermo Fisher Scientific, 15140-122, 2503-081 and 15630080), human FGF (20 ng ml^–1^) (R&D Systems, 233-FB), human EGF (10 ng ml^–1^), human IGF-I (10 ng ml^–1^) (Peprotech, AF-100-15 and 100-11) and heparin (50 μg ml^–1^) (Sigma-Aldrich, H3149-50KU). HUVECs (Thermo Fisher Scientific, C0035C) were cultured with Medium 200 supplemented with low-serum growth supplement (Thermo Fisher Scientific, M200500 and S00310) or a endothelial growth medium 2 kit with the provided supplements (EGF, FGF, IGF, but no VEGF) (PromoCell, C-22111). KG1 HSC-like AML cells (obtained from Chute’s Laboratory, originally from the American Type Culture Collection) and MKPL1 cells (obtained from K. Li, originally from DSMZ) were cultured in RPMI with 10% FBS, 1% penicillin–streptomycin and 1% l-glutamine. Cell lines were not authenticated and not tested for mycoplasma contamination.

### Cell sorting and flow cytometry for HSC assays

For identification and sorting of human HSPCs, single-cell suspensions were stained with different combinations of antibodies against human CD34, CD38, CD90, GPI-80, EPCR, ITGA3 and CD45RA. Dead cells were excluded with 7AAD or DAPI. Cells were assayed on a BD LSR Fortessa with Diva (v.8) software and analysed using FlowJo software (Tree Star). Cell sorting was performed using a BD FACS Aria II.

### RNA-seq for human haematopoietic tissues and cell lines

For RNA-seq of human haematopoietic tissues, cells were isolated as described above. The different populations were sorted directly into RLT based on the surface markers shown in Supplementary Table 10. RNA was extracted using a RNeasy Micro kit (Qiagen, 74004) and all RNA samples were sent to the CIRM consortium^65^ for library preparation and sequencing at the Next Generation Sequencing Core Facility at the Salk Institute for Biological Studies. Sequencing libraries were prepared using an Ovation RNA-seq system V2 kit (NuGEN). Paired-end 150 bp sequencing was performed on an Illumina HiSeq 4000. Alignment to both the human genome (hg37) and comprehensive genome annotation from Gencode (v.36) was performed using STAR^66^. Data were normalized to FPKM.

For KG1 and E4EC cell lines, total RNA was extracted using a RNeasy Mini kit (Qiagen, 74104) and the library was constructed using a KAPA Stranded RNA-Seq kit with a RiboErase kit. Different adapters were used for multiplexing samples in one lane. Sequencing was performed on an Illumina HiSeq 3000 for SE 1 × 50 run. Data quality check was done using Illumina SAV. Demultiplexing was performed using Illumina Bcl2fastq (v.2.19.1.403) software. Alignment to the human genome (hg38) was performed using STAR^66^. The hg38–Ensembl Transcripts release 101 gtf was used for gene feature annotation. Data were normalized to reads per kilobase million (RPKM).

### Lentiviral vectors for *MYCT1* shRNA-mediated KD and OE

For *MYCT1* KD shRNA experiments, pLKO lentiviral vectors from the MISSION TRC library (Millipore-Sigma) containing a puromycin resistance gene with or without GFP were used: TRCN00000135691 (KD1), TRCN00000137125 (KD2) and pLKO control (SHC001). shRNA sequences are provided in Supplementary Table 11.

For *MYCT1* OE, human *MYCT1* was cloned from human FL HSPC full-length cDNA into the constitutive FUGW lentiviral vector (Addgene, plasmid 14883, from D. Baltimore). *MYCT1* cDNA with a C-terminal V5 tag was inserted downstream and in-frame with the GFP sequence with the synthetic addition of a P2A sequence between the two ORFs using PfuUltra II Fusion High Fidelity DNA polymerase (Agilent 600670). Cloning primers are provided in Supplementary Table 11.

### Lentiviral production of shRNA and OE vectors and transduction

For lentiviral production, 15–20 million 293T cells were plated with DMEM (Thermo Fisher Scientific, 11995065) without antibiotics the day before transfection. Cells were transfected with 16 μg deltaR8.2 packaging plasmid, 8 μg VSVG-envelope plasmid, 20 μg of the lentiviral vector plasmid of choice and 132 μl of Turbo DNAfectin 3000 (Lambda Biotech, G3000) in Opti-MEM (Thermo Fisher Scientific, 31985070). At 6–8 h after transfection, the medium was changed to fresh complete DMEM. At 72 h after transfection, the supernatant was filtered and concentrated by ultracentrifugation, and pelleted viruses were resuspended in 200 μl SFEM or M199 (100× concentrated) and stored at −80 °C.

HSPCs were thawed, sorted and pre-stimulated for 24 h before transduction. Transduction was performed with retronectin-bound spin infection. In brief, non-treated plates were coated with Retronectin (Takara, T100B) solution overnight at 4 °C, blocked with 2% BSA in DPBS and washed with DPBS. Virus (multiplicity of infection of 50–100) was added to the coated plate and centrifuged at 2,000*g* for 2 h at 32 °C. The supernatant was removed, HSPCs were plated at a density of about 25,000 cells per cm^2^ with Lentiboost (1:100) (Sirion Biotech, SB-A-LF-900-01) and centrifuged again at 800*g* for 90 min at room temperature. The culture medium was changed the day after transduction. For shRNA lentiviral vectors, HSPCs were selected with puromycin (1 μg ml^–1^) (Invivogen, ant-pr-1), starting 24 h after transduction. ECs were transduced by directly adding concentrated virus to cultured cells (10 μl ml^–1^).

### Assessment of *MYCT1* expression by RT–qPCR during HSPC culture, after *MYCT1* KD and OE or after sorting of dextran fractions

RNA isolation was performed using a RNeasy Micro or Mini kit (Qiagen, 74004 and 74104) with an additional DNase step using the manufacturer’s protocol. cDNAs were prepared using a High-Capacity cDNA Reverse Transcription kit (Thermo Fisher Scientific, 4368814). qPCR for *GAPDH* and *MYCT1* was performed using LightCycler 480 SYBR Green I master mix (Roche, 4707516001) on a LightCycler 480 (Roche). Primers are presented in Supplementary Table 11.

### HSPC culture and expansion assays

Human HSPCs were cultured in serum-free conditions with StemSpan SFEM II (StemCell Technologies, 9655) supplemented with 1% penicillin–streptomycin, 1% l-glutamine, human FLT3-L (100 ng ml^–1^), human TPO (50 ng ml^–1^), human SCF (125 ng ml^–1^) (Thermo Fisher Scientific, 15140122, 25030081, PHC9411, PHC9513 and PHC2113), human low-density lipoprotein (10 μg ml^–1^), SR1 (500 nM) and UM171 (35 nM) (StemCell Technologies, 2698, 72352 and 72914). Cells were cultured at 37 °C and 5% CO_2_, re-plated as necessary to maintain a cell density of ≤1 × 10^6^ per ml, and half medium changes were performed every other day. For HSPC expansion assays, cells were analysed by flow cytometry every 5–14 days. Absolute counting beads (Thermo Fisher Scientific, C36950) were used to determine cell numbers and to calculate expansion rates. For co-culture with ECs, E4EC cells were plated in standard medium (M199 with serum and cytokines, see above) the day before adding HSPCs. When co-cultured with HSPCs, the standard supplemented StemSpan medium was used.

### Proliferation and cell cycle assays

Dye dilution (CellTrace) proliferation assays and monitoring of single cell divisions were performed for control and *MYCT1* KD or OE HSPCs 72–96 h after transduction. To monitor single cell divisions, transduced HSPCs (CD34^+^CD38^–^CD90^+^) were sorted into single cells in 96-well round-bottom plates 72–96 h after transduction. The cells in each well were visually counted every 24 h for 96 h.

For dye dilution proliferation assays, cells were resuspended at a concentration of 1 × 10^6^ cells per ml with warm DPBS with 5% BSA and 5  μM CellTrace Violet (Thermo Fisher Scientific, C34571) and incubated at 37 °C for 15 min protected from light. The cells were then washed with 5× volume of cold DPBS 5% BSA, incubated for 5 min at 37 °C and resuspended in pre-warmed culture medium and incubated for at least 10 min at 37 °C. The cells were stained for HSC surface markers as described above and sorted for CD34^+^CD90^+^ and a high narrow peak of CellTrace Violet. Cells were analysed by FACS immediately after sorting and after 48 h in culture.

### Colony-forming assays

CB HSPCs were transduced with control or *MYCT1* OE vectors. At 72–96 h after transduction, 180–300 sorted HSPCs (CD34^+^CD38^–^CD90^+^GFP^+^) were plated with methylcellulose-based medium containing recombinant cytokines for human cells (Methocult, StemCell Technologies, H4435, contains SCF, IL-3, IL-6, EPO, G-CSF and GM-CSF) onto two 35 mm dishes (90–150 HSPCs per plate in duplicates). Colonies were counted and morphologically assessed after 15 days.

### HSPC transplantation assays after *MYCT1* KD and FACS quantification of human multilineage engraftment

Engraftment ability of human *MYCT1* KD and control HSPCs was assessed by transplanting equal numbers of sorted HSPCs into 9–11-week-old immunodeficient NSG mice. Sorted FL or CB CD34^+^CD38^–^CD90^+^ HSPCs were transduced 24 h after sorting with *MYCT1* shRNA (KD1) or control lentiviruses, selected with puromycin, re-sorted 72 h after transduction and transplanted into female NSG mice. Around 10,000 HSPCs (CD34^+^CD38^–^CD90^+^GPI80^+^) from FL or 5,000 cells (CD34^+^CD38^–^CD90^+^) from CB were injected retro-orbitally into 6–16-week-old female NSG mice (Jackson Laboratory). Mice were pre-conditioned by sublethal irradiation (2.75 Gy) 24 h before transplantation or 25 mg kg^–1^ busulfan treatment (intraperitoneal injection) 24 and 48 h before transplantation.

Human haematopoietic engraftment was quantified 6 and 12 weeks after transplantation by BM biopsy. NSG mice from *MYCT1* KD transplantation experiments were further analysed 24 weeks after transplantation by collecting cardiac blood, spleen and BM of euthanized mice. Human engraftment was evaluated by FACS after staining for human and mouse CD45, and multilineage engraftment was determined by the detection of human myeloid (CD14 or CD66b), B lymphoid (CD19) and T lymphoid or other (CD3, CD4 and/or CD8) markers within human CD45^+^ cells (see Supplementary Table 9 for antibody details). The HSPC compartment was also evaluated using CD34 and CD38 markers.

### HSPC transplantation assays with *MYCT1 OE* and FACS quantification of human multilineage engraftment

Engraftment ability of human *MYCT1* OE and control HSPCs was assessed by transplanting equal numbers of sorted HSPCs or their progeny into immunodeficient NBSGW mice and evaluating human engraftment at 12 weeks, a time point when multilineage engraftment levels in this mouse model have been reported to peak^43,67,68^. To assess whether MYCT1 improves or alters the function of immunophenotypic HSPCs after short-term culture, CB HSPCs were transduced with *MYCT1* OE or control lentiviral vectors, sorted and transplanted 96 h after transduction into female or male 6–17-week-old NBSGW mice (Jackson Laboratory) without irradiation. About 500 or 2,500 sorted HSPCs (CD34^+^CD38^–^CD90^+^GFP^+^) were resuspended and injected as described above.

To assess the effects of sustained MYCT1 expression on the function of HSPC progeny after 15 days in culture, sorted CB HSPCs were transduced with *MYCT1* OE or control lentiviral vectors, GFP^+^ cells were re-sorted 72 h after transduction and further cultured for 10 additional days (total of 15 days in culture) and transplanted at limiting dilution doses. The number of cells reported (50, 250, 500, 2,500 or 10,000 cells) is the number of sorted GFP^+^ cells that were used to initiate the expansion cultures, the progeny of which was transplanted per mouse at day 15. Male and female NBSGW mice (6–15 weeks old; Jackson Laboratory) were used as recipients.

For *MYCT1* OE experiments, mice were considered to be engrafted if human CD45 was ≥0.1% of the total mouse and human haematopoietic compartment, and were considered to have multilineage engraftment if they displayed >0.01% of myeloid (CD14 or CD66b), B lymphoid (CD19) and T lymphoid or other (CD3, CD4 and/or CD8) cells among the total mouse and human haematopoietic compartment. Erythroid engraftment was determined by the presence of CD71^+^GlyA^+^ cells. Frequency of reconstituting units was estimated from the total engraftment data with extreme LDA^41^ including all mice from all replicates.

All transplanted mice were included in the analysis unless they died before the particular experimental time point (1 out of 9 mice transplanted with *MYCT1* KD HSPCs, 2 out of 82 mice transplanted with control vector HSPC for the OE experiment). All studies and procedures involving mice were conducted in compliance with ethical regulations and were approved by the UCLA Animal Research Committee (protocol 2005-109). Mice were housed with a 12-h light–dark cycle (6:00 to 18:00), at 20–26 °C ambient temperature and 30–70% humidity. No sample size calculations were performed a priori. Sample size for transplantation experiments was decided based on previous publications with similar HSC transplantation experiments by our laboratory or others. Allocation of mice to experimental groups was randomized, which ensured equal distribution of ages and sexes. Investigators were not blinded.

### scRNA-seq for uncultured and cultured *MYCT1* OE and KD HSPCs

CB HSPCs were sorted after thawing and sequenced directly (uncultured) or transduced with control, *MYCT1* KD and *MYCT1* OE vectors and re-sorted (CD34^+^CD38^–^CD90^+^GFP^+^) 72 h after transduction. For scRNA-seq, single-cell suspensions in DPBS 0.04% Ultrapure BSA (Thermo Fisher Scientific, AM2616) were used. A Chromium single cell instrument (10x Genomics) was used for the generation of single-cell gel beads in emulsion. scRNA-seq libraries were prepared by using a Chromium single-cell 3′ library and gel bead kit v3 (10x Genomics). Sequencing was performed on an Illumina NovaSeq 6000 system. CellRanger mkfastq (v.2.1.1) was used to generate the fastq files, the CellRanger count was mapped to the human reference genome (refdata-cellranger-GRCh38-1.2.0) and the digital expression matrix was extracted from the ‘filtered_gene_bc_matrices’ folder output.

### scRNA-seq for HSPCs with different levels of endocytosis

CB HSPCs were sorted (CD34^+^CD38^–^CD90^+^) after thawing and cultured for 4 days (96 h). HSPCs were then stained normally, placed back in culture to equilibrate for 1 h and incubated with fluorescent dextran as described below for 30 min (see section ‘FACS-based endocytosis assays’). HSPCs (CD34^+^CD38^–^CD90^+^EPCR^+^) were sorted into three fractions based on their dextran signal (20% lowest, 20% medium and 20% highest). The cells were prepared for scRNA-seq and sequenced as described above. The data from the three fractions were balanced when aggregating the samples to equilibrate the sequencing depth.

### scRNA-seq data analysis

The scRNA-seq data were analysed using the R package Seurat (v.3.1.2). Cells with fewer than 100 unique molecular identifiers (UMIs) or more than 10% mitochondrial expression were removed from further analysis. Raw counts were normalized (Seurat function NormalizeData), variable genes identified (FindVariableGenes), expression values were scaled and centred in the dataset and the number of UMIs regressed against each gene (ScaleData). Principal component analysis, t-SNE and UMAP were used to reduce the dimensions of the data.

To investigate the differences between control, *MYCT1* KD and *MYCT1* OE HSCs, cells with more than 1 count for HLF were selected (HLF^+^), the differentially expressed genes between HLF^+^ cells in the different samples (control, KD and OE) were obtained using the Seurat FindMarkers function using the DESeq2 (v.1.26.0) test. Functional enrichment analysis was performed using both gProfiler^69^ and PathfindR^70^ (v.2.3.0). For gProfiler, the significant upregulated or downregulated genes (adjusted *P* < 0.05) were inputted ordered by log fold change and run as ordered query using the following sources: GO terms, KEGG signalling pathways, Reactome (REAC) and Wikipathway (WP), regulatory motif matches (TRANSFAC, TF), and protein complexes (CORUM). For PathfindR, all genes obtained from the Findmarkers analysis were inputted, including their log fold change and adjusted *P* value, and GO was used as the source.

HLF^+^ cells from control, KD and OE HSPCs were analysed separately from uncultured and HLF^–^ cells by generating a Seurat subsample. We compiled a HSC gene set by including human HSC-associated genes from the dataset from ref. ^18^, as well as the genes included in the curated HSC cell type signature gene sets from the GSEA database^33^ (datasets from ref. ^34^, ref. ^36^ and ref. ^35^). The final HSC gene set was matched with the lists of differentially expressed genes to determine whether HSC-associated genes were differentially expressed in our HSC scRNA-seq dataset. Gene modules were compiled for ETS transcription factors and for the relevant functional categories by including all genes belonging to the particular GO term (obtained from QuickGO^71^; https://www.ebi.ac.uk/QuickGO/annotations). Scores were calculated using AddModuleScore with default parameters, and significance was calculated using Wilcoxon rank-sum test. The module scores and the expression patterns of selected genes are shown using the DotPlot function. The results of functional enrichment analysis, the gene lists used for module scores and the *P* values for each module are provided in Supplementary Table 3.

MYCT1-regulated programmes from *MYCT1* OE and KD datasets were compared with the scRNA-seq data of HSCs with different levels of endocytosis. Module scores and significance were calculated for the dextran scRNA-seq data and plotted using AddModuleScore and DotPlot as performed for *MYCT1* KD and OE samples. The *P* values for each module are provided in Supplementary Table 7.

### RNA-seq analysis of published mouse HSC datasets

MYCT1-regulated programmes from *MYCT1* OE and KD datasets were compared to a previously published RNA-seq data of mouse HSPCs^41^ (Gene Expression Omnibus (GEO) identifier GSE175400). This dataset compared gene expression profiles of the progeny of single LT-HSCs after 28 days of culture in medium containing PVA, 10 ng ml^–1^ SCF and 100 ng ml^–1^ TPO. The resulting progeny had been sorted for phenotypic HSCs (EPCR^+^LIN^–^SCA1^+^KIT^–^, named ELSK) and the remaining non-ELSK cells and transplanted in parallel to RNA-seq. Therefore, the sorted progeny were further classified as repopulating and non-repopulating^41^.

### FACS-based mitochondrial assays

Mitochondrial membrane potential and mitochondrial ROS were quantified using TMRE (Abcam, ab113852) and MitoSOX (Thermo Fisher Scientific, M36008), respectively. HSPCs transduced with control, *MYCT1* KD or *MYCT1* OE lentiviral vectors were incubated for 20 min at 37 °C with 50 nM of TMRE or 5 μM of MitoSOX. Because HSCs can efflux MitoSOX dyes, 50 μM of verapamil was added during incubation^72^. DAPI was included to discriminate dead cells.

### FACS-based endocytosis assays

HSPC or ECs transduced with control, *MYCT1* KD or *MYCT1* OE lentiviral vectors were incubated with low-molecular-weight fluorescent dextran specific for the detection of endocytosis (40 μg ml^–1^; 10 kDa, pHrodo Red Dextran, Thermo Fisher Scientific, P10361) with or ECgreen (1:1,000; Dojindo, E296) for 30 min at 37 °C. After incubation, cells were immediately placed on ice, washed with ice-cold DPBS with 5% FBS and resuspended into single-cell suspension for FACS analysis. 7AAD was included to discriminate dead cells.

### Cell fractionation

Cell fractionation was performed on 2 × 10^6^ KG1 cells overexpressing MYCT1–V5 using a Subcellular Protein Fractionation kit for cultured cells (Thermo Fisher Scientific, 78840) and following the manufacturer’s instructions with the addition of wash steps in between fractions.

### Immunofluorescence

KG1 cells overexpressing MYCT1–V5 were spun on slides at 200*g* for 10 min, fixed with 4% paraformaldehyde (Electron Microscopy Sciences, 15710) for 10 min, permeabilized with 0.1% Triton X-100 (Sigma T9284) for 5 min, rinsed twice with DPBS and blocked (DPBS with 0.5% BSA, 5% donkey serum (Jackson Immunoresearch, 017-000-121), 5% goat serum (Abcam, ab7481) and 0.1% Triton) for 30 min. Primary antibodies were prepared in blocking solution and incubated overnight at 4 °C. Slides were washed 4 times with permeabilizing solution, and secondary antibodies prepared in blocking buffer were incubated for 1 h at room temperature. Slides were washed 4 times, stained with DAPI (Miltenyi Biotec, 130-111-570) solution (1:1,000) for 10 min, rinsed with DPBS and mounted with ProLong Gold antifade mountant (Thermo Fisher Scientific, P10144). For immunofluorescence in CB HSPCs transduced with *MYCT1* OE vector, GFP^+^ cells were sorted 72 h after transduction and spun on poly-lysine (Sigma-Aldrich P8920) coated slides. Staining was performed the same way, with the addition of ImageIT signal enhancer (Thermo Fisher Scientific, I36933) for 30 min at room temperature before the blocking step. For antibody details, see Supplementary Table 9. Images were acquired with a Zeiss LSM880 confocal with Zen black software (v.14.0.29.201) at ×40 (for KG1 cells) or at ×63 using high-resolution Airyscan technology (for CB HSPCs) and processed and analysed for colocalization using Imaris (v.9.7.2).

### MYCT1–V5 IP

KG1 or E4EC cells were washed twice with ice-cold DPBS and lysed with RIPA buffer containing protease inhibitors (Thermo Fisher Scientific, 78429) by rotating the tubes for 30 min. Cell debris was removed by centrifugation for 15 min at 14,000*g* and collecting the supernatant. Protein concentration was quantified by BCA. Next, 1–2 mg of protein, together with 1–2 mg of protein G beads and 4–8 μg of V5 antibody (Thermo Fisher Scientific, 10004D and R960-25) were incubated overnight with rotation. The beads were washed 3 times with wash buffer (150 mM NaCl, 50 nM Tris pH 7.5) with 0.5% NP-40 and 5 times with wash buffer without NP-40 for 10 min each. Beads were eluted in 80 μl urea 8 M in 100 mM Tris-HCl pH 8 digestion buffer by shaking at 25 °C for 30 min. All steps including centrifugation were performed at 4 °C unless otherwise stated. If used for western blotting, the proteins were eluted in RIPA buffer (Sigma-Aldrich, R0278) with Laemmli (Bio-Rad 1610747) containing β-mercaptoethanol (Thermo Fisher Scientific, 21985023), and 4% of the total lysate was used for input.

If the samples were used for MS, protein disulfide bonds from the eluted IP samples were subjected to reduction using 5 mM Tris (2-carboxyethyl) phosphine for 30 min, and free cysteine residues were alkylated by 10 mM iodoacetamide for another 30 min. Samples were diluted with 100 mM Tris-HCl at pH 8 to reach a urea concentration of less than 2 M then digested sequentially with Lys-C and trypsin at a 1:100 protease-to-peptide ratio for 3 and 18 h, respectively. After addition of formic acid to 5% (v/v), samples were desalted using C18 tips (Thermo Fisher Scientific, 87784) and dried in a SpeedVac vacuum concentrator and reconstituted in 5% formic acid for LC–MS/MS processing.

### Phosphoproteomics in E4EC cells after *MYCT1* KD

E4EC cells were grown in 15 cm dishes, transduced with control or *MYCT1* KD vectors (5 dishes per condition) and selected with puromycin. At 72 h after transduction, the cells were washed twice with cold DPBS and collected by scraping. The whole cell pellets were washed twice more and resuspended in 150 μl digestion buffer of 8 M urea, 100 mM Tris-HCl pH 8, 1 mM MgCl_2_, 100 µl each of phosphatase inhibitor cocktails (Abcam, ab201112) and protease inhibitors (GoldBio AEBSF, Pepstatin A GoldBio P-020-25, Leupeptin GoldBio L-010-25) followed by reduction, alkylation and drying as described above. For enrichment of phosphorylated peptides, the dried peptides were enriched using Fe-NTA enrichment columns (Thermo Fisher Scientific, A32992) before LC–MS/MS processing.

### MS, LC–MS/MS processing and data quantification

The peptide mixtures were loaded onto a 25 cm-long, 75-μm inner diameter fused-silica capillary, packed in-house with bulk 1.9 μM ReproSil-Pur beads with 120 Å pores as previously described^73^. Peptides were analysed using a 140-min water–acetonitrile gradient delivered by a Dionex Ultimate 3000 UHPLC (Thermo Fisher Scientific) operated initially at 400 nl min^–1^ flow rate with 1% buffer B (acetonitrile solution with 3% DMSO and 0.1% formic acid) and 99% buffer A (water solution with 3% DMSO and 0.1% formic acid). Buffer B was increased to 6% over 5 min, at which time the flow rate was reduced to 200 nl min^–1^. A linear gradient from 6% to 28% buffer B was applied to the column over the course of 123 min. The linear gradient of buffer B was further increased to 28–35% for 8 min followed by a rapid ramp-up to 85% for column washing. Eluted peptides were ionized using a Nimbus electrospray ionization source (Phoenix S&T) by application of a distal voltage of 2.2 kV. All label-free MS data were collected using data-dependent acquisition on Orbitrap Fusion Lumos Tribrid mass spectrometer (Thermo Fisher Scientific) with a MS1 resolution of 120,000 followed by sequential MS2 scans at a resolution of 15,000.

Label-free quantification was performed using the MaxQuant software package^74^ (v.2.5.0.0). The EMBL Human reference proteome (UP000005640 9606) was utilized for all database searches. Statistical analysis of MaxQuant output data was performed with the artMS Bioconductor package (v.1.4.2), which performs the relative quantification of protein abundance using the MSstats Bioconductor package (default parameters). Intensities were normalized across samples by median-centring the log_2_-transformed MS1 intensity distributions. The abundance of proteins missing from one condition but found in more than two biological replicates of the other condition for any given comparison were estimated by imputing intensity values from the lowest observed MS1 intensity across samples, and *P* values were randomly assigned to those between 0.05 and 0.01 for illustration purposes.

### Data analysis of MYCT1-interacting proteins from IP–MS

For the interactome IP–MS, the results were first filtered using the Contaminant Repository for Single Epitope tag IP–MS^75^, and proteins enriched in all biological replicates of MYCT1–V5 samples compared with the controls were selected. To generate the protein–protein association networks, STRING^51^ (v.11.5) was used, with high-confidence settings and *k* means clustering (https://string-db.org/cgi/input). GO, pathway analysis and CORUM protein complex analysis was performed using gProfiler^69^ (https://biit.cs.ut.ee/gprofiler/gost).

### Data analysis for phosphoproteomics

For the phosphoproteomics experiment, protein-centric pathway analysis (Reactome, WikiPathways and KEGG) was performed using gProfiler^69^ for the proteins with increased or decreased phosphorylation (log_2_ fold change ≥ 0.58 or ≤–0.58, 1.5-fold and 0.66-fold, respectively, and *P* value < 0.05). Site-centric relative kinase activity prediction was performed with KSEA^55^ (v.1.0; https://casecpb.shinyapps.io/ksea) using all the identified phospho-sites as input and the following settings: dataset from PhosphoSitePlus + NetworkKIN, NetworkKIN score cutoff=2. Site-centric pathway and perturbation analysis was performed using PTM-SEA^56^ (v.PTMsigDB v.1.9.0) with the default parameters and a minimum overlap of two for pathways or five for perturbations.

### FACS analysis of receptor internalization

Low-passage primary HUVECs or CB HSPCs cells transduced with control or *MYCT1* KD vectors were starved overnight for 16 h by replacing the regular growth medium with starvation medium (growth medium without FBS, FGF, EGF and IGF1 for HUVEC cells, without SCF, FLT3-L and TPO, but containing SR1 and UM171 for HSPCs), and re-stimulated with EGF 10 ng ml^–1^ (Peprotech, AF-100-15) or SCF (125 ng ml^–1^, Thermo Fisher Scientific, PHC2113), respectively, for the indicated time points. Cells were immediately placed on ice, washed and stained. HUVECs were stained with EGFR antibody whereas HSPCs were stained with CD34, EPCR and KIT antibodies. Cells were washed and analysed by flow cytometry. DAPI was included to discriminate dead cells.

### Western blotting for signalling activation

E4EC cells or low-passage primary HUVECs transduced with control or *MYCT1* KD vectors were collected 72 h after transduction or starved overnight for 16 h by replacing the regular growth medium (see the section ‘Cell lines’) with starvation medium (growth medium without FBS, FGF, EGF and IGF1), and re-stimulated with human EGF 10 ng ml^–1^ (Peprotech, AF-100-15) or regular growth medium containing serum and cytokines for the indicated time points, which were made to coincide with 72 h since transduction. CB HSPCs were lysed 72–96 h after transduction with control, *MYCT1* KD or *MYCT1* OE vectors. Cells were lysed in RIPA buffer containing protease and phosphatase inhibitors, and protein quantification was performed using a BCA protein quantification kit (Thermo Fisher Scientific, 78440 and 23227). The lysates were prepared with Laemli containing β-mercaptoethanol and the proteins were denatured for 10 min at 95 °C. Approximately 4 μg of protein was loaded per well. Western blot images were acquired using a BioRad Chemidoc Touch Imaging System and quantified using ImageLab (v.6.0.1).

For antibody details see Supplementary Table 9. For uncropped and unprocessed scans of the western blots see Supplementary Information Figs. 1–5.

### Reporting summary

Further information on research design is available in the Nature Portfolio Reporting Summary linked to this article.

## Online content

Any methods, additional references, Nature Portfolio reporting summaries, source data, extended data, supplementary information, acknowledgements, peer review information; details of author contributions and competing interests; and statements of data and code availability are available at 10.1038/s41586-024-07478-x.

### Supplementary information

Supplementary Figures Supplementary Figs. 1–5

Reporting Summary

Supplementary Table 1 **Engraftment after transplantation of control and** ***MYCT1*** **KD HSPCs**. Quantification of human haematopoietic engraftment (human CD45^+^) in NSG mice transplanted with equal number of sorted CB or FL HSPCs transduced with control or *MYCT1* shRNA. The age at transplantation and type of conditioning are shown. Quantification shows bone marrow engraftment at 6, 12 and 24 weeks after transplantation, and spleen and blood 24 weeks after transplantation, as well as frequency of myeloid and lymphoid populations relative to human CD45 in BM 24 weeks after transplantation.

Supplementary Table 2 **Differentially expressed genes in MYCT1**^**+**^ **and MYCT1**^**–**^ **uncultured HLF**^**+**^ **HSCs from scRNA-seq. a** Differentially expressed genes between MYCT1-positive (MYCT1pos) and -negative (MYCT1neg) HLF^+^ HSCs from uncultured CB using DESeq2, which tests for differential expression based on a model using the negative binomial distribution. Wald test *P* values and Benjamini and Hochberg-adjusted *P* values are shown.

Supplementary Table 3 **Differentially expressed genes in** ***MYCT1*** **KD and OE HLF**^+^ **HSCs and functional enrichment analysis from scRNA-seq**. **a,b**, Differentially expressed genes between *MYCT1* KD and control HLF^+^ HSCs **(a)** or *MYCT1* OE and control HLF^+^ HSCs (**b**) using DESeq2, which tests for differential expression based on a model using the negative binomial distribution. Wald test *P* values and Benjamini and Hochberg-adjusted *P* values are shown. **c-f,** Functional enrichment analysis for the genes significantly (*P*adj < 0.05) up or downregulated in KD (**c,d**) or OE (**e,f**) compared with control performed using gProfiler (Raudvere et al. 2019), which performs g:SCS multiple testing correction method applying significance threshold of 0.05. Data include GO terms for molecular function (GO:MF), biological process (GO:BP), and cellular compartment (GO:CC), signalling pathway analysis using KEGG, Reactome (REAC) and Wikipathway (WP), regulatory motif matches (TRANSFAC, TF), and protein complexes (CORUM). **g,h,** Functional enrichment analysis for the dysregulated genes in *MYCT1* KD (**g**) or *MYCT1* OE (**h**) compared with control HSCs performed using PathfindR GO (Ulgen et al. 2019), which provides the lowest and highest Bonferroni adjusted enrichment *P* values of the given term over all iterations. **i,** Gene lists used to generate module scores of functional properties, and their source. **j,** Gene list and sources for the genes included in the HSC gene set. **k,** *P* values calculated using Wilcoxon rank-sum test for the different module scores in *MYCT1* KD or *MYCT1* OE HSCs compared with control. **l,** Synonyms for the gene names used as input to gProfiler or in the gene modules.

Supplementary Table 4 **Engraftment after transplantation of control and** ***MYCT1*** **OE HSPCs 96** **h after transduction.** Quantification of human haematopoietic engraftment (human CD45^+^) in NBSGW mice transplanted with low and medium doses of sorted CB HSPCs transduced with control or *MYCT1* OE vectors. Quantification shows total human BM engraftment (human CD45^+^), engraftment of differentiated populations (myeloid, lymphoid, erythroid and megakaryocytic), HSPCs (CD34^+^CD38^–^) and HPCs (CD38^+^) 12 weeks after transplantation. Frequency of differentiated populations relative to human CD45 in BM is also shown.

Supplementary Table 5 **Engraftment after transplantation of the progeny of control and** ***MYCT1*** **OE HSPCs at day** **15 in culture**. Quantification of human haematopoietic engraftment (human CD45^+^) in NBSGW mice transplanted with the progeny after 15 days in culture of different doses of CB HSPCs transduced with control or *MYCT1* OE vectors. Quantification shows total human BM engraftment (human CD45^+^) and engraftment of differentiated populations (myeloid, lymphoid, erythroid and megakaryocytic), HSPCs (CD34^+^CD38^–^) and HPCs (CD38^+^) 12 weeks after transplantation. Frequency of differentiated populations relative to human CD45 in BM is also shown.

Supplementary Table 6 **MYCT1 interactome from IP–MS. a,** Number of peptides specifically detected in KG1 cells in the MYCT1 IP compared with control. All 6 replicates from *n* = 3 independent experiments are shown. **b,** Functional enrichment analysis for the MYCT1 interactome in KG1 was performed using gProfiler (Raudvere et al. 2019), which performs g:SCS multiple testing correction method applying significance threshold of 0.05. Data include GO terms for molecular function (GO:MF), biological process (GO:BP), and cellular compartment (GO:CC), signalling pathway analysis using KEGG, Reactome (REAC) and Wikipathway (WP), regulatory motif matches from Transfac (TF), and protein complexes (CORUM). **c,** Number of peptides specifically detected in E4EC cells in the MYCT1 IP compared with control. Two technical replicates from *n* = 1 experiment are shown. **d,** Functional enrichment analysis for the MYCT1 interactome in E4EC cells performed as in **b**. **e,** Average number of peptides of the MYCT1 interactors identified in KG1 and E4EC cells and their gene expression in logRPKM in ECs and HSPCs from human 5–6-week AGM (aorta-gonad-mesonephros), yolk sac (YS), placenta (PL), second trimester FL, CB and adult BM.

Supplementary Table 7 **Differentially expressed genes in endocytosis-low and endocytosis-high HLF**^**+**^ **HSCs and module scores from scRNA-seq. a,** Number of HSPCs (CD34^+^CD38^–^CD90^+^EPCR^+^) and HLF^+^ cells for each endocytosis fraction sequenced for scRNA-seq. **b,** Differentially expressed genes between endocytosis-low and endocytosis-high HLF^+^ HSCs cultured for 96 h. Calculated using DESeq2, which tests for differential expression based on a model using the negative binomial distribution. Wald test *P* values and Benjamini and Hochberg-adjusted *P* values are shown. **c,** *P* values calculated using Wilcoxon rank-sum test for the different module scores in endocytosis-low or endocytosis-mid compared with endocytosis-high.

Supplementary Table 8 **Phosphoproteomic profiling of ECs (E4EC) after control or** ***MYCT1*** **KD. a,b,** Differentially phosphorylated sites between control and *MYCT1* KD1 (**a**) or KD2 (**b**). *P* values adjusted using the approach by Benjamini and Hochberg. *P* values and adjusted *P* values are provided in the default ArtMS output (see Methods). **c,d,** Functional enrichment analysis for the proteins with increased (**c**) or decreased (**d**) phosphorylation in *MYCT1* KD compared with control performed using gProfiler (Raudvere et al. 2019), which performs g:SCS multiple testing correction method applying significance threshold of 0.05. Data include GO terms for molecular function (GO:MF), biological process (GO:BP), and cellular compartment (GO:CC), signalling pathway analysis using KEGG, Reactome (REAC) and Wikipathway (WP), and protein complexes (CORUM). **e,** Selected terms from the functional enrichment analysis shown in Extended Data Fig. 11e. **f,g,** Inferred relative kinase activity after *MYCT1* KD compared with control was calculated using kinase substrate enrichment analysis (KSEA, Wiredja et al. 2017) for KD1 (**f**) and KD2 (**g**). m denotes the total number of phosphosite substrates identified for the specified kinase. The KSEA output provides the kinase score based on a *z*-score transformation. The *P* value is determined by assessing the one-tailed probability of having a more extreme score than the one measured, followed by a Benjamini and Hochberg false discovery rate correction for multiple hypothesis testing. **h,** Perturbation and pathway scores for *MYCT1* KD 1 and 2 compared with control calculated using PTM-SEA (Krug et al. 2019, v.PTMsigDB v.1.9.0). The PTM-SEA output provides normalized enrichment scores (NES) and *P* values (calculated using 10,000 permutations) corrected for multiple hypothesis testing by Benjamini–Hochberg correction.

Supplementary Table 9 **Reagents. a,** Antibodies used for flow cytometry. **b,** Antibodies used for western blot and immunofluorescence. **c,** General reagents and kits. **d,** Reagents and materials used for proteomics.

Supplementary Table 10 **Sorting strategy for haematopoietic tissues for RNA-seq analysis**. Markers used for sorting endothelial and haematopoietic populations from embryonic, fetal and post-natal haematopoietic tissues for RNA-seq analysis.

Supplementary Table 11 **Primers and sequences. a,** Sequences for shRNA knockdown of *MYCT1*. **b,** Cloning primers used to generate the *MYCT1* OE vector. **c,** Sequence of the primers used for SyBR Green RT–qPCR of *MYCT1* and *GAPDH*.

### Source data

Source Data Fig. 1

Source Data Fig. 2

Source Data Fig. 3

Source Data Fig. 4

Source Data Fig. 5

Source Data Extended Data Fig. 1

Source Data Extended Data Fig. 2

Source Data Extended Data Fig. 3

Source Data Extended Data Fig. 4

Source Data Extended Data Fig. 5

Source Data Extended Data Fig. 6

Source Data Extended Data Fig. 7

Source Data Extended Data Fig. 8

Source Data Extended Data Fig. 9

Source Data Extended Data Fig. 10

Source Data Extended Data Fig. 11

Source Data Extended Data Fig. 12

## Data Availability

The RNA-seq and scRNA-seq datasets generated during the current study are available at the GEO under the accession codes GSE233478 (bulk RNA-seq for human haematopoietic populations), GSE232360 (bulk RNA-seq for KG1 and E4EC), GSE232361 (scRNA-seq of *MYCT1* OE and KD) and GSE254857 (scRNA-seq of endocytosis fractions). The MS and phospho MS datasets are available at PRIDE under the accession code PXD042257. There are no restrictions on data availability. Published data from ref. ^41^ are available under GSE175400. Published data are available in table S1 of ref. ^28^, table S3 of ref. ^19^ and table S3 of ref. ^9^. Published data from ref. ^10^ are available at the GEO under accession GSE111483. Source data are provided with this paper.
