# Supplementary material for: MYCT1 controls environmental sensing in human haematopoietic stem cells
Source: Nature. 2024 Jun 5;630(8016):412–20. doi: 10.1038/s41586-024-07478-x (PMC11168926; doi:10.1038/s41586-024-07478-x)
Supplement: Supplementary file 1 — Supplementary Figs. 1–5 [file 41586_2024_7478_MOESM1_ESM.pdf]

nature portfolio

https://doi.org/10.1038/s41586-024-07478-x

Supplementary information

MYCT1 controls environmental sensing in human haematopoietic stem cells

In the format provided by the authors and unedited

Supplementary Fig 1. Corresponds to Fig. 5i

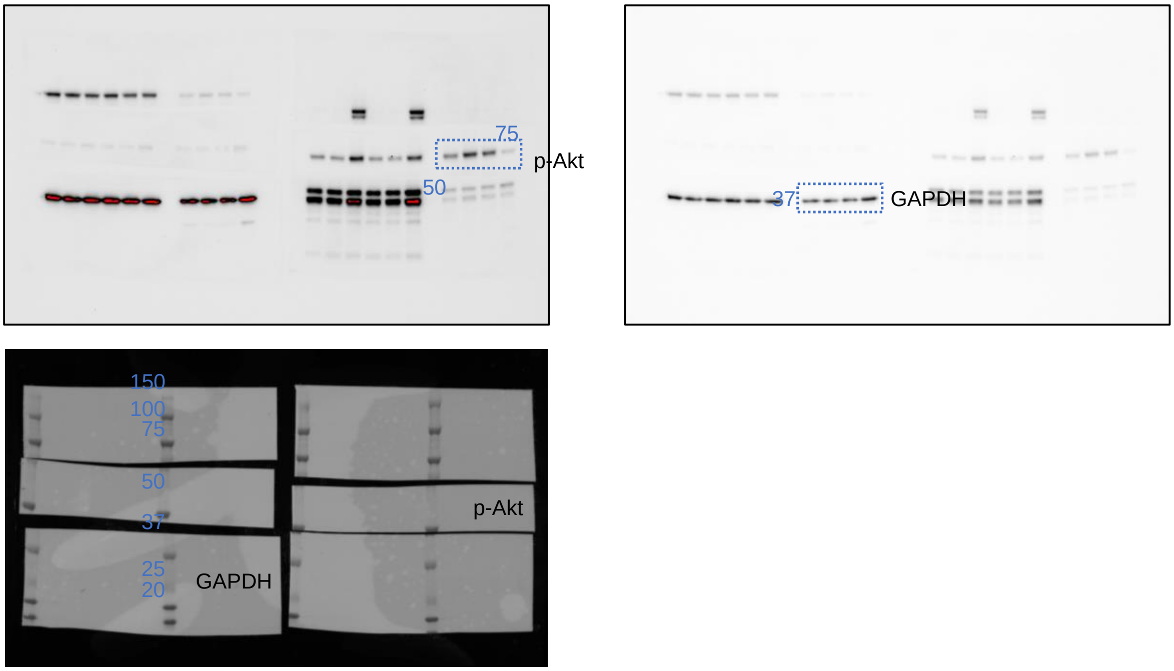

pAKT and GAPDH were run on separate gels. GAPDH is a sample processing control.
Different exposure times for the same membranes are shown

Supplementary Fig 2. Corresponds to Extended Data Fig. 2b

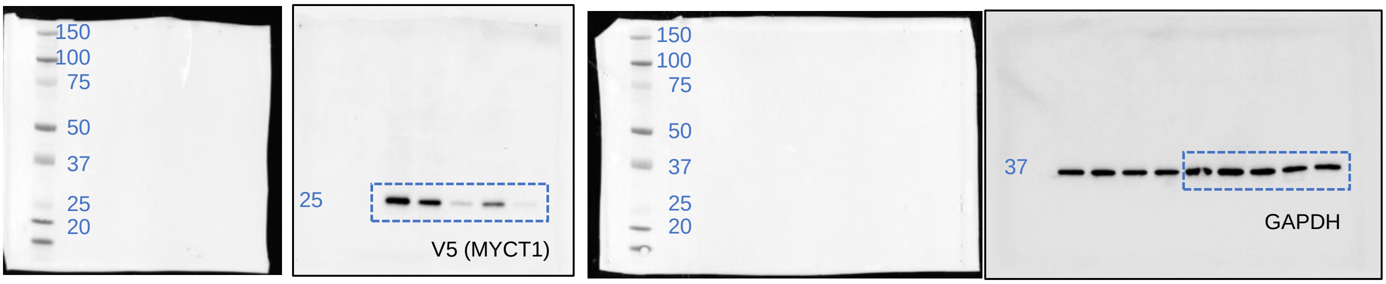

V5 and GAPDH were run on separate gels, GAPDH was run together with other samples. GAPDH is a sample processing control.

Supplementary Fig 3. Corresponds to Extended Data Fig 9c

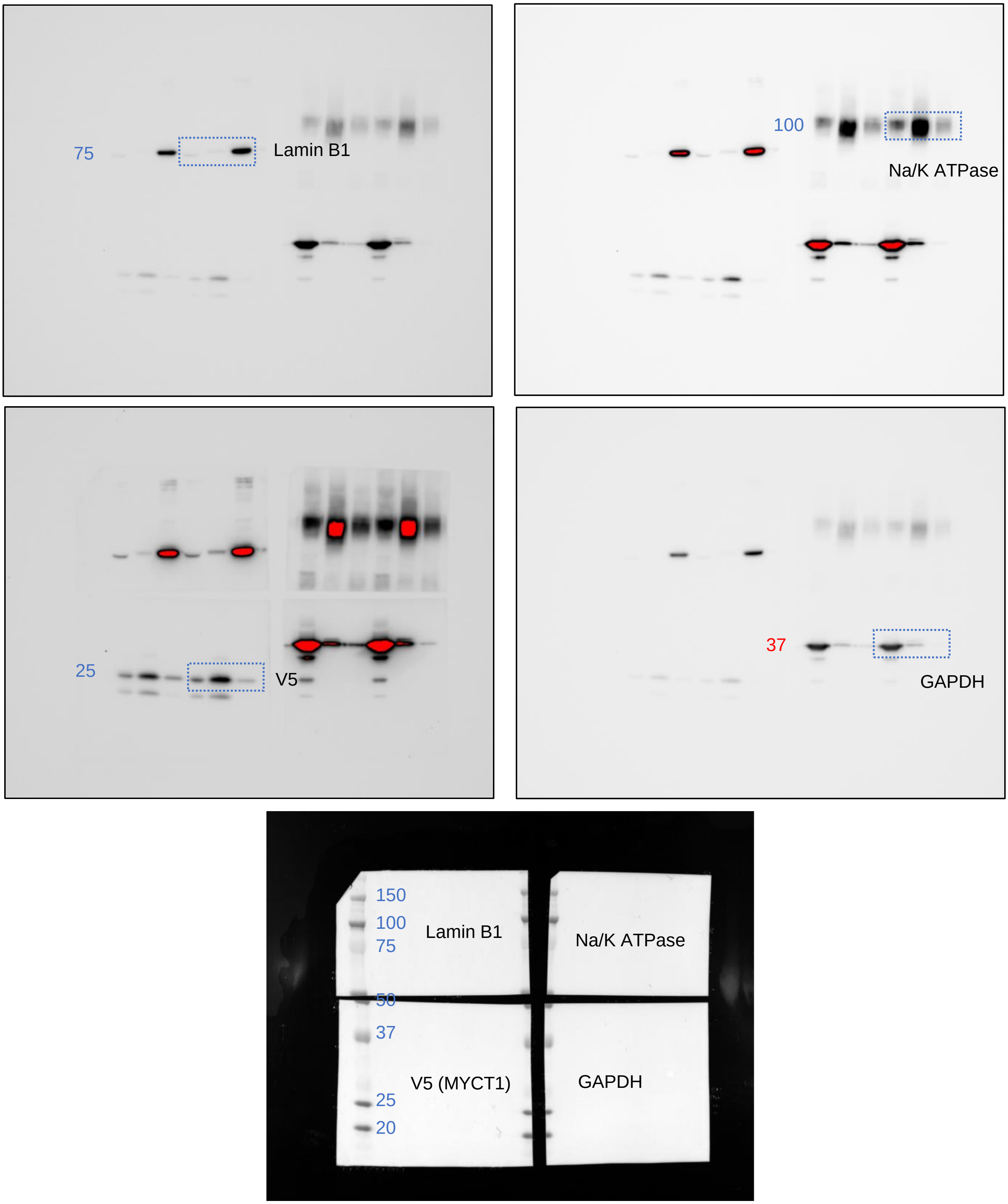

LaminB1 and V5(MYCT1) were run on one gel, Na/K ATPase and GAPDH were run on another gel. The experiment is a fractionation experiment, therefore Lamin B1, GAPDH, and Na/K ATPase are fractionation controls to assess the purity of the fractions

Supplementary Fig 4. Corresponds to Extended Data Fig 9g

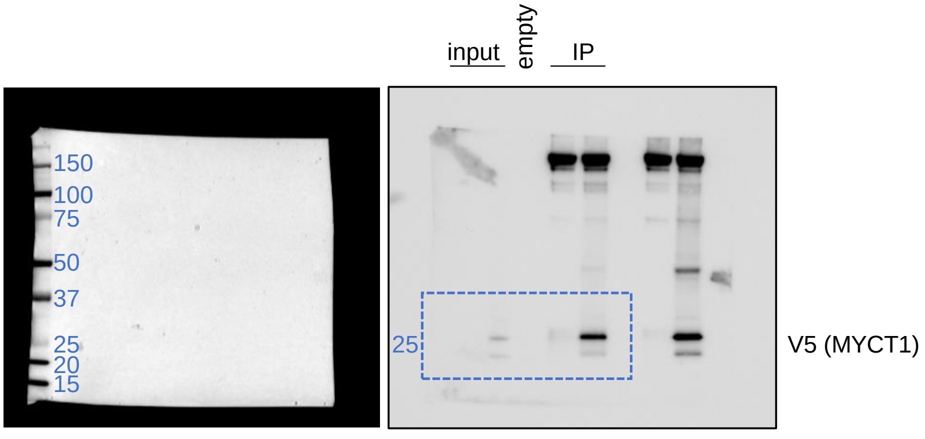

Supplementary Fig 5. Corresponds to Extended Data Fig. 12

a Corresponds to Extended Data Fig. 12b

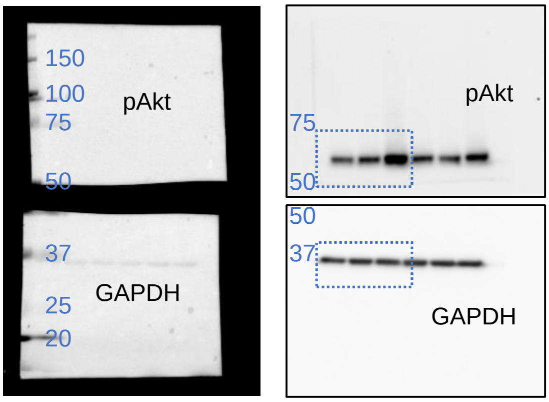

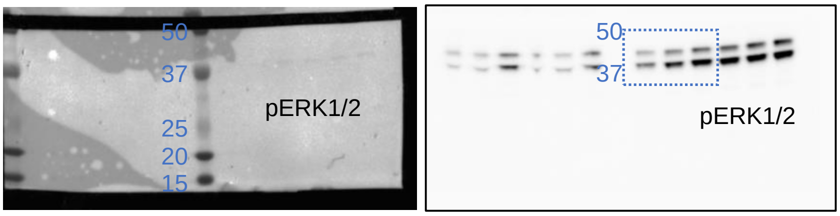

pAKT and GAPDH were run on the same gel, pERK1/2 on a different gel. GAPDH is a loading control for pAKT and a sample processing control for pERK1/2.

b Corresponds to Extended Data Fig. 12e

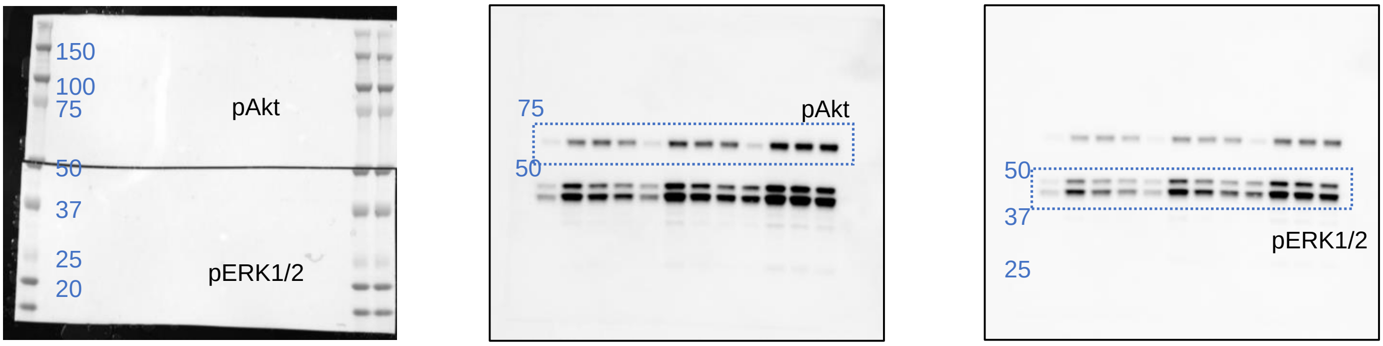

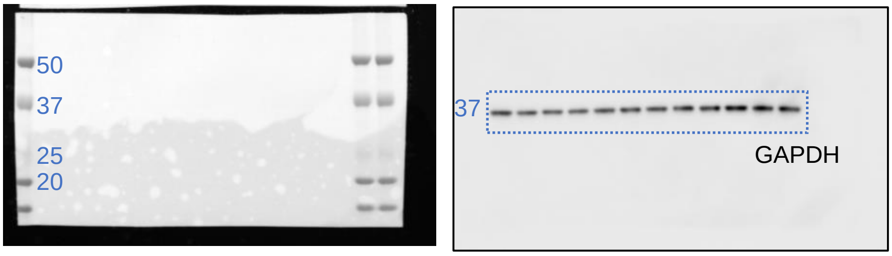

pAKT and pERK1/2 were run on the same gel, GAPDH was run on a different gel. GAPDH is a sample processing control.
Different exposure times for the same membranes are shown

c Corresponds to Extended Data Fig. 12j

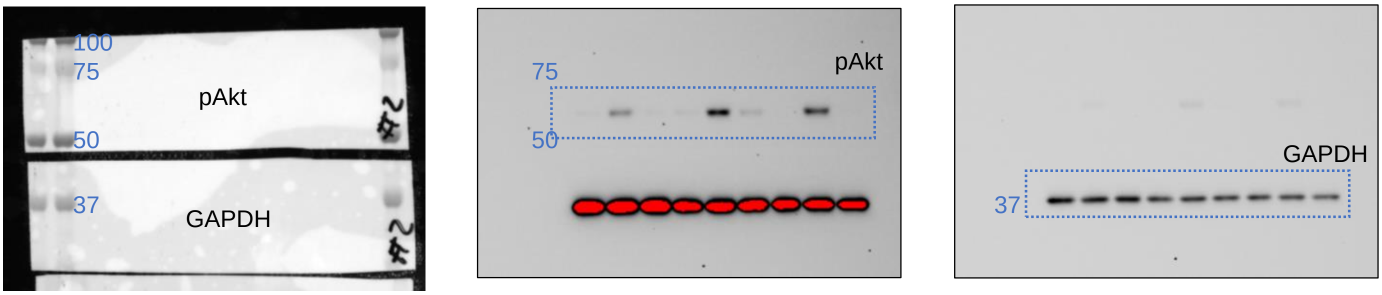

pAKT and GAPDH were run on the same gel. GAPDH is a loading control.
Different exposure times for the same membranes are shown
