# Supplementary material for: MYCT1 controls environmental sensing in human haematopoietic stem cells
Source: Nature. 2024 Jun 5;630(8016):412–20. doi: 10.1038/s41586-024-07478-x (PMC11168926; doi:10.1038/s41586-024-07478-x)
Supplement: Supplementary file 2 — Reporting Summary [file 41586_2024_7478_MOESM2_ESM.pdf]

nature portfolio

Corresponding author(s): Hanna K. A. Mikkola, Júlia Aguadé-Gorgorió

Last updated by author(s): April 12, 2024

Reporting Summary

Nature Portfolio wishes to improve the reproducibility of the work that we publish. This form provides structure for consistency and transparency in reporting. For further information on Nature Portfolio policies, see our Editorial Policies and the Editorial Policy Checklist.

Statistics

For all statistical analyses, confirm that the following items are present in the figure legend, table legend, main text, or Methods section.

n/a	Confirmed	
☐	☒	The exact sample size (*n*) for each experimental group/condition, given as a discrete number and unit of measurement
☐	☒	A statement on whether measurements were taken from distinct samples or whether the same sample was measured repeatedly
☐	☒	The statistical test(s) used AND whether they are one- or two-sided<br>*Only common tests should be described solely by name; describe more complex techniques in the Methods section.*
☒	☐	A description of all covariates tested
☒	☐	A description of any assumptions or corrections, such as tests of normality and adjustment for multiple comparisons
☐	☒	A full description of the statistical parameters including central tendency (e.g. means) or other basic estimates (e.g. regression coefficient) AND variation (e.g. standard deviation) or associated estimates of uncertainty (e.g. confidence intervals)
☐	☒	For null hypothesis testing, the test statistic (e.g. *F*, *t*, *r*) with confidence intervals, effect sizes, degrees of freedom and *P* value noted<br>*Give P values as exact values whenever suitable.*
☒	☐	For Bayesian analysis, information on the choice of priors and Markov chain Monte Carlo settings
☒	☐	For hierarchical and complex designs, identification of the appropriate level for tests and full reporting of outcomes
☐	☒	Estimates of effect sizes (e.g. Cohen's *d*, Pearson's *r*), indicating how they were calculated

Our web collection on statistics for biologists contains articles on many of the points above.

Software and code

Policy information about availability of computer code

Data collection	No software was used for single cell data acquisition. Diva v8 (BD) was used for flow cytometry acquisition. Zen black v14.0.29.201 (Zeiss) was used for immunofluorescence acquisition. All label-free mass spectrometry data were collected using data dependent acquisition on Orbitrap Fusion Lumos Tribrid mass spectrometer (Thermo Fisher Scientific). qPCR: Lightcycler 480 Roche software v1.5.1.62. Bulk RNAseq: Illumina HiSeq 3000
Data analysis	Flow cytometry: FlowJo v10.8.1 (Tree Star Inc.). Immunofluorescence: Imaris v9.7.2. Single cell RNAseq: 10x Genomics CellRanger v2.1.1, Seurat v3.1.2., DESeq2 v1.26.0, PathfindR v2.3.0 and gProfiler. Bulk RNAseq: Illumina Bcl2fastq v2.19.1.403 for demutiplexing, STAR for alignment. Mass spectrometry: MaxQuant v2.5.0.0, artMS v1.4.2, STRING v11.5. Phospho mass spectrometry: KSEA v1.0 (relative kinase activity), PTM-SEA (pathway and perturbation analysis, PTMsigDB v1.9.0). Western Blot quantification: BioRad ImageLab 6.0.1. Limiting dilution analysis: ELDA. Graph plotting and statistical analysis: Graphpad Prism v8

For manuscripts utilizing custom algorithms or software that are central to the research but not yet described in published literature, software must be made available to editors and reviewers. We strongly encourage code deposition in a community repository (e.g. GitHub). See the Nature Portfolio guidelines for submitting code & software for further information.

Data

Policy information about availability of data

All manuscripts must include a data availability statement. This statement should provide the following information, where applicable:

- Accession codes, unique identifiers, or web links for publicly available datasets
- A description of any restrictions on data availability
- For clinical datasets or third party data, please ensure that the statement adheres to our policy

The RNAseq and single cell RNAseq datasets generated during the current study are available in the GEO under the accession codes GSE233478 (bulk RNAseq for human hematopoietic populations), GSE232360 (bulk RNAseq for KG1 and E4EC), GSE232361 (single cell RNAseq of MYCT1 OE and KD), and GSE254857 (scRNAseq of endocytosis fractions). The Mass spectrometry and phosho mass spectrometry datasets are available in PRIDE under the accession code PXD042257. There are no restrictions on data availability.
Published data from Che el al. are available under GSE175400 in https://www.ncbi.nlm.nih.gov/geo/query/acc.cgi?acc=GSE175400.
Published data from García-Prat et al. are available in the original publication Table S1 https://www.sciencedirect.com/science/article/pii/S1934590921002885.
Published data from Tomellini et al. are available in the original publication Table S3 https://www.sciencedirect.com/science/article/pii/S2211124719308642.
Published data from Magnusson et al. are available in the original publication Table S3 https://journals.plos.org/plosone/article?id=10.1371/journal.pone.0053912.
Published data from Calvanese et al. are available under GSE111483 in https://www.ncbi.nlm.nih.gov/geo/query/acc.cgi?acc=GSE111483.

Research involving human participants, their data, or biological material

Policy information about studies with human participants or human data. See also policy information about sex, gender (identity/presentation), and sexual orientation and race, ethnicity and racism.

Reporting on sex and gender	Findings apply to both sexes. Sex-based analysis were not performed, and cord blood from both male and female donors was used. Our cord blood biobank contains an equivalent representation of both male (≈45%) and female (≈55%) samples, but the data is not available for all human samples.<br>Since the tissues used are either embryonic/foetal or cord blood from at least 34-week births, gender is not applicable.
Reporting on race, ethnicity, or other socially relevant groupings	Race, ethnicity, and other social information is not available for the used biological material. Social categorization is not used in our study.
Population characteristics	No other covariate-relevant population characteristics such as genotypic information or past and current diagnosis and treatment categories were obtained or collected.
Recruitment	Embryonic/foetal tissues were obtained from elective pregnancy terminations. The decision to terminate pregnancy had occurred prior to consent for tissue donation. The tissues from elective pregnancy terminations and were obtained from University clinic of Tubingen, UCLA or from Family Planning Associates through UCLA CFAR core. Cord blood samples were obtained from Cedars-Sinai in Los Angeles. The tissue sources may introduce biases in terms of socio-economic status or ethnicity of the population served by the particular hospitals. We don't expect this to impact our findings. The tissues were obtained without links to any personal identifiers.
Ethics oversight	This study uses human fetal and post-natal tissues but does not use Human Subjects as all the tissues are discarded materials without personal identifiers. UCLA IRB determined that this research does not constitute research on Human Subjects.

Note that full information on the approval of the study protocol must also be provided in the manuscript.

Field-specific reporting

Please select the one below that is the best fit for your research. If you are not sure, read the appropriate sections before making your selection.

☒ Life sciences ☐ Behavioural & social sciences ☐ Ecological, evolutionary & environmental sciences

For a reference copy of the document with all sections, see nature.com/documents/nr-reporting-summary-flat.pdf

Life sciences study design

All studies must disclose on these points even when the disclosure is negative.

Sample size	The sample size for transplantation experiments was decided based on prior publications with similar HSC transplantation experiments by our laboratory or others, including PMID: 31776511, 31340144.
Data exclusions	In scRNAseq analysis, cells with number of features <100 and percent of mitochondrial reads >0.1 were excluded, based on standard pre-established criteria. In the analysis of MYCT1 expression (RNAseq) in different populations across ontogeny, one sample of CLP1 cells from foetal liver was excluded because it was contaminated with endothelial cells (Fig 1A). In the western blot experiments of phosphoAKT in human HSPC (Fig 5i), one experiment in KD cells was excluded because it was an outlier from the multiple experiments done in the same (n=3 shown in the paper) or similar conditions (4 more experiments done with other equivalent methods or conditions).

Replication	The number of replicates for each experiment is indicated in figure legends. Replicates were identically or similarly designed.<br><br>All experiments for which representative figures are shown (western blots, FACS plots, immunofluorescence)are accompanied by quantification graphs showing all replicates and appropriate statistics.<br><br>Key findings in the loss of function experiments were successfully reproduced with human HSCs from different developmental stages (fetal liver and cord blood). All experiments with human cord blood HSPCs were successfully replicated with different donors. All attempts at replication were successful with the exception of the experiment noted above in "Data Exclusions".
Randomization	Allocation of mice to experimental groups was random, but equal distribution of ages and sexes was ensured. Cells were split equally and randomly assigned into experimental groups.
Blinding	Investigators were blinded for the morphological assessment of colony formation assays. For all other experiments, investigators were not blinded. Data collection for all experiments was automated (e.g. flow cytometry, sequencing etc.) and data interpretation was based on appropriate controls rather than subjective assessment by investigators.

Reporting for specific materials, systems and methods

We require information from authors about some types of materials, experimental systems and methods used in many studies. Here, indicate whether each material, system or method listed is relevant to your study. If you are not sure if a list item applies to your research, read the appropriate section before selecting a response.

Materials & experimental systems

n/a	Involved in the study	
☐	☒	Antibodies
☐	☒	Eukaryotic cell lines
☒	☐	Palaeontology and archaeology
☐	☒	Animals and other organisms
☒	☐	Clinical data
☒	☐	Dual use research of concern
☒	☐	Plants

Methods

n/a	Involved in the study	
☒	☐	ChIP-seq
☐	☒	Flow cytometry
☒	☐	MRI-based neuroimaging

Antibodies

Antibodies used

For FACS:
Antibody - Clone - Company - Cat number - Dilution used
CD14-V500 M5E2 BD Biosciences 561391 1:50
CD19-BV605 SJ25C1 BD Biosciences 562653 1:25
CD34-APC 581 eBiosciences 555824 1:20
CD34-APCCy7 581 BioLegend 343514 1:50
CD34-BV605 563 BD Biosciences 745105 1:25 (engraftment), 1:100 (cell culture)
CD34-BUV395 581 BD Biosciences 563778 1:50
CD38-PECy7 HIT2 BD Biosciences 560677 1:50
CD3-PECy7 SK7 BioLegend 344816 1:50
CD41a-BV510 HIP8 BD Biosciences 563250 1:50
CD45-BV711 HI30 BioLegend 304050 1:50
CD4-APC S3.5 Life Technologies MHCD0405 1:50
CD66b-BV421 G10F5 BD Biosciences 562940 1:50
CD71-AF647 DF1513 Santa Cruz Biotechnology sc-7327 AF647 1:50
CD8-PE 3B5 Beckman IM0452U 1:50
CD90-APC 5E10 BD Biosciences 559869 1:50
CD90-BV421 5E10 BD Biosciences 562556 1:50
CD90-FITC 5E10 BD Biosciences 555595 1:50
EPCR-APC RCR-401 BioLegend 351906 1:16
EPCR-APCvio 770 REA337 Miltenyi Biotec 130-125-847 1:16
GlyA-PE GA-R2 (HIR2) BD Biosciences 340947 1:50
GPI80-PE 3H9 MBL bio D087-5 1:50
mCD45-APCCy7 30-F11 BD Biosciences 557659 1:50
ITGA3-PE C3 II.1 BD Biosciences 556025 1:50
CR45RA-PerCP-Cy5.5 HI100 BioLegend 304122 1:50
EGFR-PE AY13 BioLegend 352903 1:50
cKit-PE YB5.B8 eBiosciences 12-1179-42 1:50

For western blot and immunofluorescence:
Antibody - Clone - Company - Cat number - Dilution used
Anti-Mouse IgG Secondary Antibody, HRP-linked Amersham NA931V 1:5000
Anti-Rabbit IgG Secondary Antibody, HRP-linked Amersham NA934-1ML 1:2500
Clathrin Heavy Chain D3C6 Cell Signaling Technology 4796S 1:100

Donkey Anti-Mouse IgG (H+L) Secondary Antibody, Alexa Fluor 488 Jackson Immunoresearch 715-546-150 1:200
GAPDH 14C10 Cell Signaling Technology 2118L 1:10000
Goat anti Rabbit IgG (H+L) Secondary Antibody, Alexa Fluor 647, ThermoFisher Scientific A-21245 1:200
HSP60 D6F1 Cell Signaling Technology 12165T 1:200
Lamin B1 EPR8985(B) Abcam ab133741 1:5000
Na/K ATPase EP1845Y Abcam ab76020 1:20000
phospho-AKT D9E Cell Signaling Technology 4060S 1:2000
phospho-ERK D13.14.4E Cell Signaling Technology 4370S 1:2000
Rab11 D4F5 Cell Signaling Technology 5589T 1:50
Rab5 C8B1 Cell Signaling Technology 3547T 1:200
Rab7 D95F2 Cell Signaling Technology 9367T 1:100
V5 SV5-Pk1 ThermoFisher Scientific R960-25 1:1000 (WB), 1:100 (IF)
VeriBlot for IP Detection Reagent (HRP) Abcam ab131366
GM130 EP892Y Abcam ab52649 1:100

Validation

All antibodies used for this study have been previously validated by commercial manufacturers, previous publications, and/or this study. We provide the catalog number, manufacturer and clone (where applicable) for all antibodies listed used.
For FACS:
CD14-V500 M5E2 BD Biosciences 561391, reported to recognize human CD14 (manufacturer's website)
CD19-BV605 SJ25C1 BD Biosciences 562653, reported to recognize human CD19 (manufacturer's website)
CD34-APC 581 eBiosciences 555824, reported to recognize human CD34 (manufacturer's website)
CD34-APCCy7 581 BioLegend 343514, reported to recognize human CD34 (manufacturer's website)
CD34-BV605 563 BD Biosciences 745105, reported to recognize human CD34 (manufacturer's website)
CD34-BUV395 581 BD Biosciences 563778, reported to recognize human CD34 (manufacturer's website)
CD38-PECy7 HIT2 BD Biosciences 560677, reported to recognize human CD38 (manufacturer's website)
CD3-PECy7 SK7 BioLegend 344816, reported to recognize human CD3 (manufacturer's website)
CD41a-BV510 HIP8 BD Biosciences 563250, reported to recognize human CD41a (manufacturer's website)
CD45-BV711 HI30 BioLegend 304050, reported to recognize human CD45 (manufacturer's website)
CD4-APC S3.5 Life Technologies MHCD0405, reported to recognize human CD4 (manufacturer's website)
CD66b-BV421 G10F5 BD Biosciences 562940, reported to recognize human CD66b (manufacturer's website)
CD71-AF647 DF1513 Santa Cruz Biotechnology sc-7327, reported to recognize human CD71 (manufacturer's website)
CD8-PE 3B5 Beckman IM0452U, reported to recognize human CD8 (manufacturer's website)
CD90-APC 5E10 BD Biosciences 559869, reported to recognize human CD90 (manufacturer's website)
CD90-BV421 5E10 BD Biosciences 562556, reported to recognize human CD90 (manufacturer's website)
CD90-FITC 5E10 BD Biosciences 555595, reported to recognize human CD90 (manufacturer's website)
EPCR-APC RCR-401 BioLegend 351906, reported to recognize human EPCR (manufacturer's website) and in the publication describing the use of EPCR as a human HSC surface marker (Fares et al,2017).
EPCR-APCvio 770 REA337 Miltenyi Biotec 130-125-847, reported to recognize human EPCR (manufacturer's website) and in the publication describing the use of EPCR as a human HSC surface marker (Fares et al,2017).
GlyA-PE GA-R2 (HIR2) BD Biosciences 340947, reported to recognize human GlyA (manufacturer's website)
GPI80-PE 3H9 MBL bio D087-5, reported to recognize human GPI80 (manufacturer's website) and in the publication describing the use of GPI80 as a human fetal liver HSC surface marker (Prashad et al, 2015).
mCD45-APCCy7 30-F11 BD Biosciences 557659, reported to recognize mouse CD45 (manufacturer's website)
ITGA3-PE C3 II.1 BD Biosciences 556025, reported to recognize human ITGA3 (manufacturer's website) and in the publication describing the use of ITGA3 as a human HSC surface marker (Tomellini et al,2019).
CR45RA-PerCP-Cy5.5 HI100 BioLegend 304122, reported to recognize human CD45RA (manufacturer's website)
EGFR-PE AY13 BioLegend 352903, reported to recognize human EGFR (manufacturer's website)
cKit-PE YB5.B8 eBiosciences 12-1179-42, reported to recognize human cKit (manufacturer's website)

For western blot and immunofluorescence:
Anti-Mouse IgG Secondary Antibody, HRP-linked Amersham NA931V, reported to be species specific for mouse IgG (manufacturer's website)
Anti-Rabbit IgG Secondary Antibody, HRP-linked Amersham NA934, reported to be species specific for mouse IgG (manufacturer's website)
Clathrin Heavy Chain D3C6 Cell Signaling Technology 4796S, reported recognize endogenous human Clathrin Heavy Chain (manufacturer's website)
Donkey Anti-Mouse IgG (H+L) Secondary Antibody, Alexa Fluor 488 Jackson Immunoresearch 715-546-150, reported recognize Mouse IgG (manufacturer's website). Additionally, we confirmed the antibody specificity for immunofluorescence by staining with secondary antibody without any primary antibody.
GAPDH 14C10 Cell Signaling Technology 2118L, reported recognize endogenous human GAPDH (manufacturer's website)
Goat anti Rabbit IgG (H+L) Secondary Antibody, Alexa Fluor 647, ThermoFisher Scientific A-21245, reported recognize Rabbit IgG (manufacturer's website). Additionally, we confirmed the antibody specificity for immunofluorescence by staining with secondary antibody without any primary antibody.
HSP60 D6F1 Cell Signaling Technology 12165T, reported recognize endogenous human HSP60 (manufacturer's website)
Lamin B1 EPR8985(B) Abcam ab133741, reported recognize human LaminB1 (manufacturer's website)
Na/K ATPase EP1845Y Abcam ab76020, reported recognize human Na/K ATPase (manufacturer's website)
phospho-AKT D9E Cell Signaling Technology 4060S, reported recognize endogenous human pAKT (Ser 473) (manufacturer's website)
phospho-ERK D13.14.4E Cell Signaling Technology 4370S, reported recognize endogenous human pERK1/2 (Thr202/Tyr204) (manufacturer's website)
Rab11 D4F5 Cell Signaling Technology 5589T, reported recognize endogenous human Rab11 (manufacturer's website)
Rab5 C8B1 Cell Signaling Technology 3547T, reported recognize endogenous human Rab5 (manufacturer's website)
Rab7 D95F2 Cell Signaling Technology 9367T, reported recognize endogenous human Rab7 (manufacturer's website)
V5 SV5-Pk1 ThermoFisher Scientific R960-25, reported recognize V5 tag (manufacturer's website). Additinally we validated V5 antibody for western blot, immunofluorescence and immunoprecipitation by using negative and positive cells (untransduced and MYCT1-V5 transduced cell lines).

VeriBlot for IP Detection Reagent (HRP) Abcam ab131366, reported recognize immunoblotted target protein bands without interference from denaturated IgG (manufacturer's website)
GM130 EP892Y Abcam ab52649, reported recognize endogenous human GM130 (manufacturer's website)

Eukaryotic cell lines

Policy information about cell lines and Sex and Gender in Research

Cell line source(s)	293T (from ATCC), KG1 (obtained from Dr. John Chute, originally from ATCC), E4-immortalized HUVEC (E4EC generated by and obtained from Dr. Rafii, Butler et al. 2012), HUVEC (Thermofisher Scientific), MKPL1 (Dr. Kailong Li, originally from DSMZ).
Authentication	The cell lines were not authenticated
Mycoplasma contamination	Not tested
Commonly misidentified lines (See ICLAC register)	No commonly misidentified cell lines were used

Animals and other research organisms

Policy information about studies involving animals; ARRIVE guidelines recommended for reporting animal research, and Sex and Gender in Research

Laboratory animals	For human HSC transplantation, 6-17 weeks females from the NSG (NOD.Cg-Prkdcscid Il2rgtm1Wjl/SzJ) strain or females and males from the NBSGW (NOD.Cg-KitW-41J Tyr + Prkdcscid Il2rgtm1Wjl/ThomJ) strain were used. The age, sex and strain are indicated in the legends, methods and extended data tables. Mouse housing: 12 hours light/dark cycle (6am to 6pm), temperature 20-26ºC, humidity 30-70%.
Wild animals	No wild animals were used in these studies
Reporting on sex	Mice were used for transplantation experiments of human HSCs. Findings are applicable to both sexes. For the NSG model only female NSG mice were used due to their higher efficiency of human HSC engraftment. Both male and female NBSGW mice were used for transplantation in the MYCT1 overexpression experiments, always ensuring equal distribution among control and experimental groups. Data disaggregated for sex is reported in the extended data figures and extended data tables corresponding to the transplantation experiments. Analysis of the transplantations that include both male and female mice is shown pair-wise, and similar results were observed in male and female mice, so the aggregated data was used to draw biological conclusions.
Field-collected samples	The study did not involve samples collected from the field
Ethics oversight	All experiments were carried out in accordance with ethical care guidelines set by University of California on Laboratory Animal Care. Specific protocol numbers available on request.

Note that full information on the approval of the study protocol must also be provided in the manuscript.

Flow Cytometry

Plots

Confirm that:

☒ The axis labels state the marker and fluorochrome used (e.g. CD4-FITC).

☒ The axis scales are clearly visible. Include numbers along axes only for bottom left plot of group (a 'group' is an analysis of identical markers).

☒ All plots are contour plots with outliers or pseudocolor plots.

☒ A numerical value for number of cells or percentage (with statistics) is provided.

Methodology

Sample preparation	FACS analysis was performed using single cell suspensions prepared from human fetal liver and cord blood after isolation from the tissues or culture, or obtained from NSG/NBSGW mice transplanted with human cells. Cells were stained in PBS 5% FBS.
Instrument	Cells were assayed on a BD Fortessa flow cytometer. Cell sorting was performed using a BD FACS Aria II.
Software	BD DIVA v8<br>FlowJo v10.8.1
Cell population abundance	Cell purity was checked periodically when setting up the sorting instruments, resulting in >95 % purity
Gating strategy	For both flow cytometry analysis and sorting, cells were selected for live cell scatter in FSC/SSC then for singlets in FSCh/FSCw and SSCh/SSCw. Cells were then gated for dead cell exclusion (7AAD or DAPI negative). GFP on transduced cells was gated

based on untransduced controls. Other markers were determined positive when signal was above FMO (fluorescence minus one) control.

☒ Tick this box to confirm that a figure exemplifying the gating strategy is provided in the Supplementary Information.
