# Supplementary material for: MYCT1 controls environmental sensing in human haematopoietic stem cells
Source: Nature. 2024 Jun 5;630(8016):412–20. doi: 10.1038/s41586-024-07478-x (PMC11168926; doi:10.1038/s41586-024-07478-x)
Supplement: Supplementary file 13 — Primers and sequences. a, Sequences for shRNA knockdown of MYCT1. b, Cloning primers used to generate the MYCT1 OE vector. c, Sequence of the primers used for SyBR Green RT–qPCR of MYCT1 and GAPDH. [file 41586_2024_7478_MOESM13_ESM.pdf]

Supplementary Table 11: Primers and sequences

a. Sequences for shRNA knockdown of MYCT1

KD	CAT. NUMBER	SEQUENCE
KD1	TRCN00000135691	CCTGGAACAAGCAAATTCCTT
KD2	TRCN00000137125	CAAGCAGGAGATCTAGGTCTT

b. Cloning primers used to generate the MYCT1 OE vector

NAME	SEQUENCE
P2A_MYCT1_FWD	GCGACGTGGAAGAGAACCCTGGCCCCATGCGAACACAAGTATATGAG
MYCT1_V5_REV	GGTTGGGGATGGGCTTGCCGCTAGCGGAATCTGGGAATGCCTTGATG

a. Primers used for RT-qPCR with SyBR Green

NAME	SEQUENCE
MYCT1 FWD	GACTCTCCCTTCTTCCAATA
MYCT1 REV	GGCCCACTCGAAGACTGTTA
GAPDH FWD	GGAGCGAGATCCCTCCAAAAT
GAPDHR	GGCTGTTGTCATACTTCTCATGG
